# Implementation of an antimicrobial stewardship program in Alexandria University Children’s Hospital: an interventional study

**DOI:** 10.1186/s13052-026-02236-3

**Published:** 2026-04-10

**Authors:** Sarah Elsayed Saad Refaei, Laila El-Attar, Amira Ezzat Khamis Amine, Marwa Ahmed Meheissen, Eman Hamza Hassan

**Affiliations:** 1 Alexandria University Children’s Hospital, Alexanderia, Egypt; 2 Department of Microbiology, High Institute of Public Health, Alexandria University, Alexanderia, Egypt; 3 Department of Microbiology, Faculty of Medicine, Alexandria University, Alexanderia, Egypt; 4 Department of Pediatric, Faculty of Medicine, Alexandria university, Alexanderia, Egypt

**Keywords:** Antimicrobial stewardship program, Days of therapy, Institutional guidelines, Prospective audit and feedback (PAF)

## Abstract

**Background:**

Antimicrobial resistance (AMR) represents a threat to global public health. The antibiotics’ effectiveness against a variety of infections consequently has been declined with increasing morbidity, mortality, and treatment failure. To combat this, the implementation of Antimicrobial Stewardship Programs (ASPs) is essential for slowing the spread of resistant pathogens.

**Aim of the work:**

The current study aimed to assess the outcomes following implementation of Antimicrobial Stewardship Program (ASP) at Alexandria University Children’s Hospital.

**Method:**

The study was conducted over nine months in a general pediatric ward. First, the medical records and microbiological reports were reviewed to establish tailored antibiotic guidelines. During the intervention phase, the ASP focused on prospective audits and physicians’ education. The program’s impact was evaluated through several key metrics: adherence to the guidelines, patient outcome (mortality rate and length of stay) and antibiotic consumption (expenditure, days of therapy, and treatment duration). All statistical analyses were conducted using IBM SPSS version 20.0.

**Results:**

219 patients in the preintervention phase were compared to 214 patients (post-intervention). Following ASP, the use of single antibiotics increased in post-intervention (69.6% versus 26%). During the study period, 70 interventions were required with a high acceptance rate (59%). The mean length of hospital stays decreased [10.80 (3-26) versus 12.89 (4–33) days], and both DOT/1000 patients and the average cost of antibiotics decreased (27.82% and 44.94%, respectively). Following ASP, the use of Tigecycline (-100%), Meropenem (-57.79%), and Vancomycin (-46.35%) reduced with an increase in the use of Cefotaxime (80.43%), Ceftriaxone (20.27%), and Ceftazidime (62.87%).

**Conclusion:**

The implementation of institutional guidelines along with Prospective Audit and Feedback (PAF) was associated with improvements in antibiotic utilization, particularly in the resource-limited settings.

**Clinical trial number:**

Not applicable.

**Supplementary Information:**

The online version contains supplementary material available at 10.1186/s13052-026-02236-3.

## Background

Antimicrobials are the cornerstone of modern medicine, decreasing morbidity and mortality. After penicillin’s introduction in 1928, resistance was recognized in the 1940s [1]. In 2019, the Centres for Disease Control and Prevention (CDC) reported that 2.8 million patients were infected with multidrug-resistant organisms (MDRO) in the United States yearly, and about 35,000 deaths are directly attributed to those infections. In 2022, the updated CDC report found the rate of these infections increased by 20% during the covid pandemic [2]. A systematic review of antimicrobial resistance in the Middle East detected variable percentages of carbapenem resistance among *Acinetobacter baumannii* (74.2%), E. coli (8.1%), and K. pneumoniae (15.4%). Moreover, Extended spectrum B-lactamase (ESBL)-producing *E. coli* and *Klebsiella pneumoniae* were 32.3% and 27.9%, respectively [3]. Antimicrobial resistance (AMR) is expected to cause ten million deaths globally by 2050 and increase healthcare costs by up to $1 trillion [4]. 

Inappropriate antibiotic prescription is a major contributing factor to the rise in antimicrobial resistance. The inappropriate use of antimicrobials includes the use of antibiotics to treat non-infectious conditions, using antibiotics for prolonged durations, using broad-spectrum antimicrobials when they are not needed, overprescription of antibiotics, and access to over-the-counter antibiotics. Antibiotic stewardship program (ASP) is an integrated interventions that promote better antibiotic prescription, improves patient outcomes, and decreases the emergence of antimicrobial resistance. Furthermore, it has a positive impact on hospital costs and lengths of stay [5, 6]. Implementation of ASP in low- and middle-income countries faces many challenges, including gaps in the availability of appropriate guidelines, few infectious disease training programs, limited laboratory resources, gaps in data on antibiotic use and antibiotic resistance patterns, and inexperience with data analysis. High-yield strategies are urgently needed. Strategies must be feasible and tailored to the resources available in such countries [7]. 

## Methods

### Aim of the work

The primary objective of this study was to set customized clinical guidelines for antibiotic treatment based on local antibiograms and implement antimicrobial stewardship (ASP) in a pediatric medical ward. The second objective was to compare the consumption and cost of antimicrobial agents using days of therapy (DOT) and the direct cost of antibiotics before and after the ASP program implementation.

### Study setting and population

The present received ethical approval from the Institutional Review Board of the Hight Institute of Public Health at Alexandria University. The current study was conducted in a general pediatric ward at Alexandria University Children’s Hospital The study was conducted over a nine-month period from December 2020 to August 2021. The study was carried out in three phases:

### Pre-intervention phase over three months (from december 2020 to february 2021)

During this phase, all culture results of patients admitted over a period of one year preceding the study (January 2020 to January 2021) were reviewed from the hospital’s microbiology laboratory records. The WHONET software was used in recording and analysing the institutional antibiogram to detect the prevalence of multidrug-resistant organisms and their cumulative antimicrobial susceptibility patterns to help in establishing institutional guidelines for common Pediatrics infections (supplementary material).

Patients admitted to the pediatric intensive care unit were excluded. The medical records of patients admitted during this period were reviewed, and the following data were collected: - Demographic data, principal diagnosis, number of antibiotics used, and unnecessary duplicate therapy. Antimicrobial use based on days of therapy (DOT). Outcome of the patient, length of stay, and antibiotic costs.

### Intervention phase of ASP (from march 2021 to may 2021)

The institutional guideline for the most common community-acquired infections was developed by adapting in the international guidelines to the local antibiogram (supplementary material). This initiative was supported by a comprehensive educational lecture for pediatric clinicians, covering antibiotic pharmacology, rational prescribing, risks of antibiotic resistance and the principles of ASP. Following the dissemination of this guideline, Antimicrobial Stewardship Program (ASP) was launched. This intervention centred on Prospective audit and Feedback (PAF), conducted during daily round by infectious disease specialist and clinical pharmacist [8]. This allowed for real-time adjustments to therapy based on the microbiological data. To further enhance diagnostic precision, multiplex-PCR testing for central nervous system infection (CNS) and respiratory tract infections was integrated in to the laboratory workflow, facilitating the rapid identification of viral pathogens and the subsequent reduction of unnecessary antibiotic use.

### Post-intervention phase of ASP (from june 2021 to august 2021)

Following implementation of the ASP, clinical data was collected using the same parameters as the pre-intervention phase. Furthermore, specific stewardship metrics were evaluated, including the frequency of ‘antibiotic timeouts,’ rates of inappropriate therapy, and dosing inaccuracies. The program’s impact was further quantified by calculating the number of interventions required per patient and the rate of physician adherence to the stewardship recommendations. Children with pre-existing comorbidities, and children with healthcare-associated infections were excluded from both the pre-intervention and post-intervention cohorts.

### Statistical analysis

Data was fed to the computer and analyzed using IBM SPSS software package version 20.0. Qualitative data were described using number and percentage. The Shapiro-Wilk test was used to verify the normality of distribution. Chi-square test for categorical variables, to compare between different groups and Mann Whitney to compare utilization and costs for each class of antibiotics between the two periods. For all tests, p-values of < 0.05 were considered statistically significant.

## Results

The present study included 219 children with a mean age of 38.83 ± 41.154 months were in the preintervention phase and 214 patients with a mean age of 36.00 ± 41.49 months in the postintervention phase, with a male predominance in both phases (55.7% and 52.5%, respectively). A significant difference existed (*P* = 0.028) in the indications for antibiotic prescription, central nervous system infections (CNS) (27.3%), urinary tract infections (UTI) (22.8%), and lower respiratory tract infections (20.5%) were the most common indications for antibiotic prescriptions. While, in the post-intervention phase, respiratory tract infections (32.7%) and UTIs (18.7%) were the most common indications for antibiotic prescription (Table 1). The difference in the indications is related to seasonal variations over the study period and the impact of COVID19 during the study period.

**Table 1:** Indications and number of antibiotics use in pre- and post- intervention phase

Patients’ characteristics	Pre -intervention (*n* = 219)	Post-intervention (*n* = 214)	Sig.
**Age (Mean in months ± SD)**	38.83 ± 41.154	36.00 ± 41.49	*P* = 0.472
**Sex**			
Male	122(55.7%)	115(52.5%)	*P* = 0.621
Female	96(43.8%)	99(45.2%)	
**Indications**			
Blood stream infection	27(12.3%)	25(11.7%)	
Cardiovascular system infections	5(2.3%)	2(0.9%)	
Central nervous system infections	60(27.3%)	23(10.7%)	*P* = 0.028*†
Fever of well appearing children	4(1.8%)	2(0.9%)	
Intra-abdominal infections	10(4.6%)	28(13.1%)	
Noninfectious cause	9(4.5%)	17(7.9%)	
Lower respiratory tract infections	45(20.5%)	70(32.7%)	
Skin and soft tissue infections	9(4.1%)	7(3.2%)	
Urinary tract infections	50(22.8%)	40(18.7%)	
**Number of prescribed antibiotics**			
1	57 (26.0%)	149 (69.6%)	*P* = 0.052†
2	89 (40.6%)	56 (26.2%)	
3	55 (25.1%)	6 (2.8%)	
4	16 (7.3%)	3 (1.4%)	
5	2 (0.9%)	0	
**Unnecessary duplicate therapy**	33 (15.1%)	9 (4.2%)	*P* < 0.001*

Sig:- Significance, SD: Standard deviation, Related-Samples Marginal Homogeneity Test, McNemar-Bowker Test, * Significant results *p* ≤ 0.05

Following implementation of the Antimicrobial Stewardship Program (ASP), there was a notable improvement in antibiotic prescription. Unnecessary duplicate empiric therapy showed a significant reduction from 15.1% to 4.2% (*P* < 0.001). furthermore, there was increase in monotherapy from 26% (*n* = 57) to 69.6% (*n* = 149). However, this increase in single-agent prescription was statistically insignificant (*P* = 0.052) (Table 1).

During the post-intervention period, researchers suggested 70 specific actions related to prospective audit and feedback, involving 63 (29.4%) patients. The overall acceptance of implementation of the feedback was 41% (*n* = 29). The most frequent intervention was antibiotic time-out (41.4%) followed by correction for inappropriate therapy at 28.6%. conversely, correcting inaccurate dose calculations was the least common (4%), but achieved the highest rate of acceptance (Table 2).

**Table 2:** Required intervention measures during post-intervention phase

	Post-intervention *(n = 214*)		
**ASP recommendations**			
Not required	151 (70.6%)		
Required	63* (29.4%)		
**Types of required ASP intervention measures (** *n* * = 70)*	**No.** **(%)**	**Accepted**	**Not accepted**
Antibiotic time out	29 (41.4%)	17	12
Inappropriate	20 (28.6%)	9	11
Antibiotic not indicated	17 (24.3%)	11	6
Incorrect dose calculation	4 (6%)	4	0
**Total interventions**	70 (100%)	41 (59%)	29 (41%)

* In 63 patient 70 intervention required as in some patients more than one intervention required

**Fig. 1:**
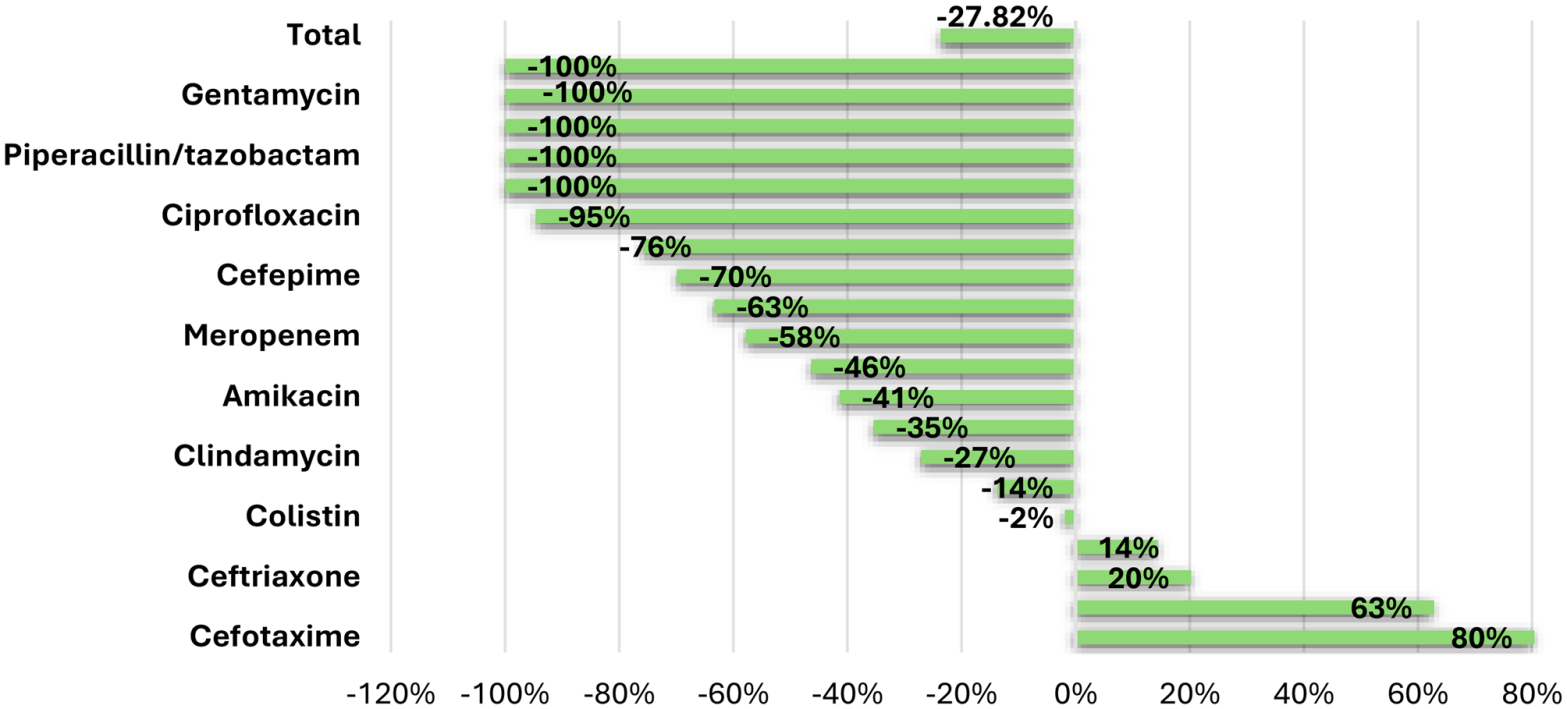
Percent Change of DOT /1000 patient days per each antibiotic before and after intervention

To evaluate the effectiveness of the ASP, we compared the antibiotic consumption- measured in total Days of Therapy (DPT) of each antibiotic and DOT per1,000 patient’s days in pre-and post-intervention phases. The results demonstrated a significant reduction in overall antibiotic utilization. In the post-intervention period, the total antibiotic days of therapy (DOT) decreased by 40.91%, highlighting the program’s success in controlling antibiotic use [Table 3; Figure 1]. The DOT/1000 patients showed a significant reduction of 27.82%. The use of broad-spectrum antibiotics decreased significantly, including a decrease in tigecycline (-100%), meropenem (-57.79%), and vancomycin (-46.35%). In contrast the use of the 3rd generation cephalosporins showed a significant increase (Table 3 Fig. 1). The use of acyclovir showed a statistically significant drop (*P* < 0.001), which decreased from 118.31 to28.56 DOT/1,000 patient days. This decline in the post-intervention phase was attributed to the integration of multiplex PCR testing, which enabled more accurate identification of pathogens in CNS infections and facilitated more targeted treatment decisions.

**Table 3:** DOT of antimicrobials and DOT/1000 patient days of studied patients in the pre and post intervention phases

Drug name	DOT (days)				% of change DOT	DOT/ 1000pt days		DOT/ 1000pt days		% of change DOT/ 1000pt days		Sig.
	Before		After			Before intervention ^a^		After intervention ^b^				
	No.	%	No.	%		No.	%	No.	%			
Tigecycline	40	0.9	0	0.0	-100.00%	14.17	0.9	0.00	0.0	-100.00%		0.001^*^
Teicoplanin	17	0.4	0	0.0	-100.00%	6.02	0.4	0.00	0.0	-100.00%		0.002^*^
Piperacillin/tazobactam	10	0.2	0	0.0	-100.00%	3.54	0.2	0.00	0.0	-100.00%		0.017^*^
Imipenem/cilastatin	20	0.5	0	0.0	-100.00%	7.08	0.5	0.00	0.0	-100.00%		0.001^*^
Gentamycin	18	0.4	0	0.0	-100.00%	6.38	0.4	0.00	0.0	-100.00%		0.001^*^
Ciprofloxacin	68	1.6	3	0.1	-95.59%	24.09	1.6	1.30	0.1	-94.61%		< 0.001^*^
Acyclovir	334	7.7	66	2.6	-80.24%	118.31	7.7	28.56	2.6	-75.86%		< 0.001^*^
Cefepime	77	1.8	19	0.7	-75.32%	27.28	1.8	8.22	0.7	-69.86%		< 0.001^*^
Linezolid	60	1.4	18	0.7	-70.00%	21.25	1.4	7.79	0.7	-63.35%		0.010^*^
Meropenem	929	21.3	321	12.5	-65.45%	329.08	21.3	138.90	12.5	-57.79%		< 0.001^*^
Vancomycin	979	22.4	430	16.7	-56.08%	346.79	22.4	186.07	16.7	-46.35%		< 0.001^*^
Amikacin	50	1.1	24	0.9	-52.00%	17.71	1.1	10.39	0.9	-41.37%		0.399
Ampicillin/sulbactam	172	3.9	91	3.5	-47.09%	60.93	3.9	39.38	3.5	-35.37%		0.385
Clindamycin	67	1.5	40	1.6	-40.30%	23.73	1.5	17.31	1.6	-27.07%		0.958
Cefoperazone/sulbactam	194	4.4	137	5.3	-29.38%	68.72	4.4	59.28	5.3	-13.74%		0.101
Colistin	61	1.4	49	1.9	-19.67%	21.61	1.4	21.20	1.9	-1.88%		0.105
Levofloxacin	79	1.8	74	2.9	-6.33%	27.98	1.8	32.02	2.9	14.42%		0.004^*^
Ceftriaxone	905	20.7	891	34.6	-1.55%	320.58	20.7	385.55	34.6	20.27%		< 0.001^*^
Ceftazidime	21	0.5	28	1.1	33.33%	7.44	0.5	12.12	1.1	62.87%		0.004^*^
Cefotaxime	262	6.0	387	15.0	47.71%	92.81	6.0	167.46	15.0	80.43%		< 0.001^*^
Total	4363		2578		-40.91%	1545.52		1115.53		-27.82%		

Sig.: Significance * Statistically significant at *p* ≤ 0.05

^a^ Patient days before intervention, 2823 days

^b^ Patient days after intervention, 2311 days

**Fig. 2:**
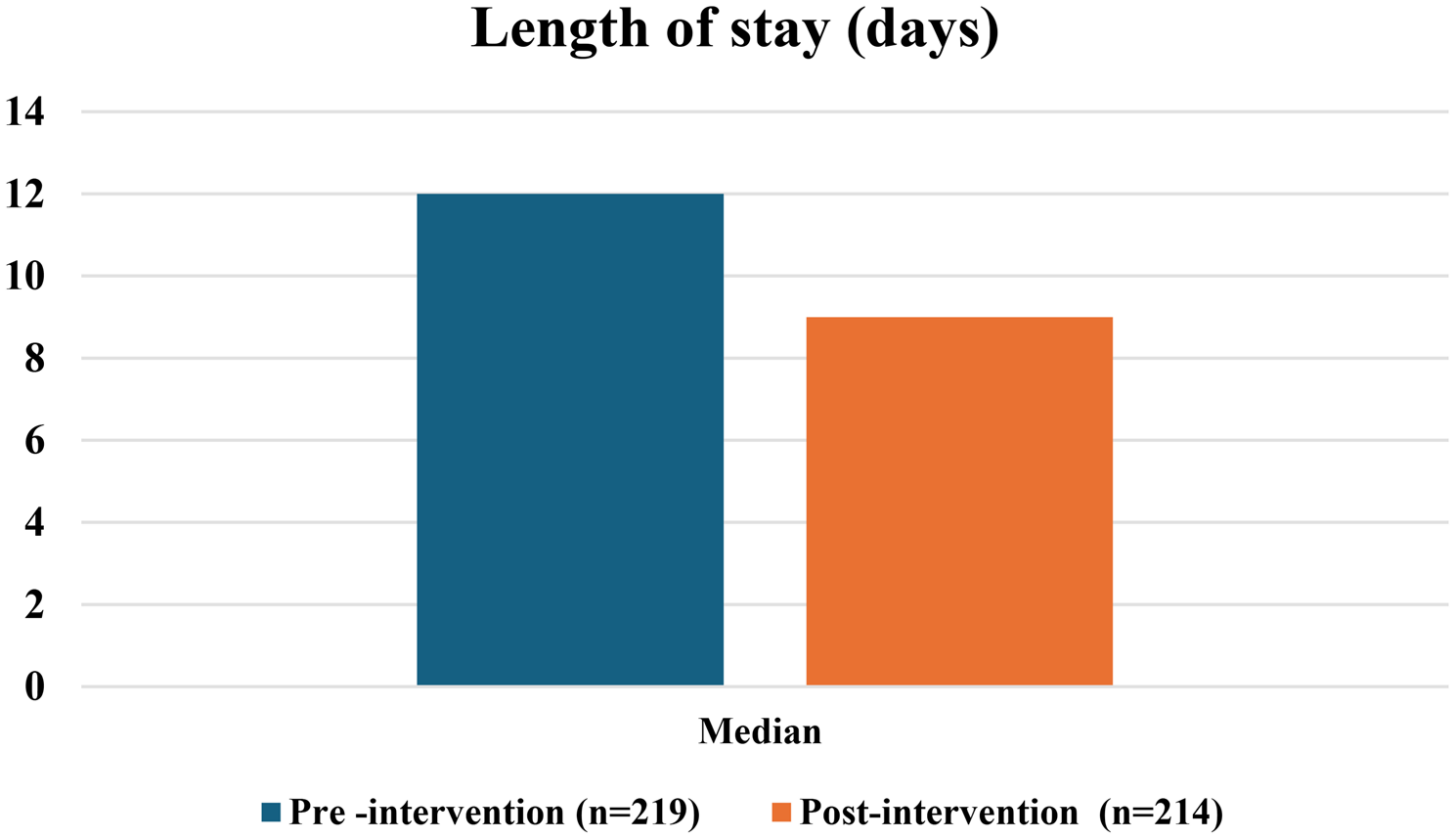
Length of stay (LOS) between the pre-and post-intervention phases

**Fig. 3:**
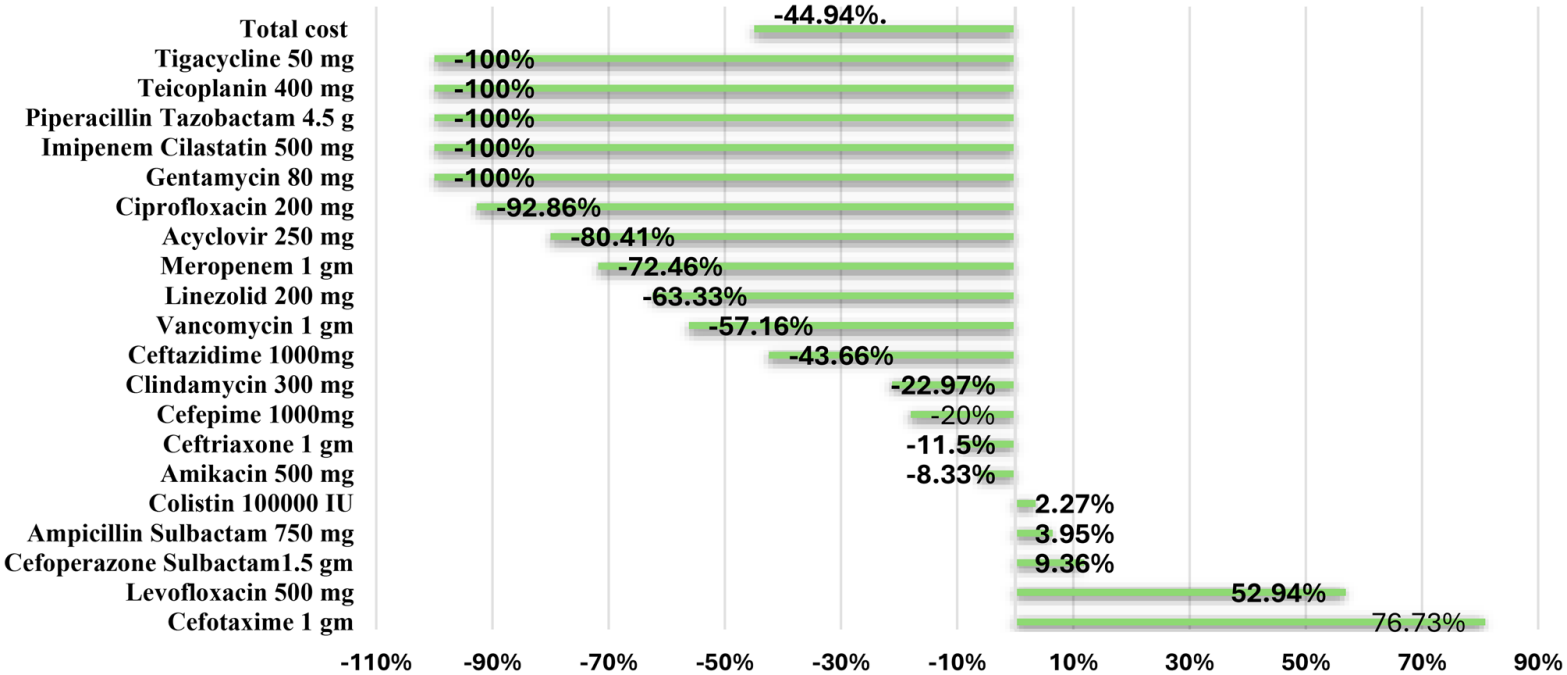
Percent change in cost of consumed antibiotics in pre- and post-intervention phases

Following implementation, the average length of hospital stays decreased significantly (*P* < 0.001) from 10.80 (3-26) to to12.89 (4–33) days in the post-intervention [Table 4 and Fig. 2]. Furthermore, the average cost of antibiotic consumed decreased by 44.94% between the two phases, which was a statistically significant (*P* < 0.001. Ciprofloxacin, acyclovir, meropenem, and vancomycin all showed a significant cost reduction (P values = 0.034, *P* < 0.001, < 0.001, and < 0.001; respectively) (Table 5; Fig. 3).

**Table 4:** Comparison of the length of stay (LOS) between the pre-and post-intervention phases

Length of stay (days)	Pre -intervention *(* *n* * = 219)*	Post-intervention *(n = 214*)	Sig.
Min. – Max.	4.0–33.0	3.0–26.0	*P* < 0.001^*^
Mean ± SD.	12.89 ± 6.13	10.80 ± 5.01	
Median	12.0	9.0	

SD: Standard deviation Sig.: Significance

* Statistically significant at *p* ≤ 0.05

**Table 5:** Average cost of consumed antibiotics of the studied patients in the pre and post intervention phases

Drug name	Consumption (vials)		Cost (L.E)		Cost (L.E) Mean ± SD.		% difference	*P*
	Pre -intervention (*n* = 219)	Post-intervention (*n* = 214)	Pre -intervention (*n* = 219)	Post-intervention (*n* = 214)	Pre -intervention (*n* = 219)	Post-intervention (*n* = 214)		
Tigecycline 50 mg	40	0	10,720	0	48.95 ± 724.39	0.0 ± 0.0	-100%	0.323
Teicoplanin 400 mg	43	0	7939.52	0	36.25 ± 536.50	0.0 ± 0.0	-100%	0.323
Piperacillin Tazobactam 4.5 gm	13	0	819	0	3.74 ± 44.39	0.0 ± 0.0	-100%	0.162
Imipenem Cilastatin 500 mg	58	0	6133.5	0	28.01 ± 320.68	0.0 ± 0.0	-100%	0.162
Gentamycin 80 mg	9	0	27	0	0.12 ± 1.36	0.0 ± 0.0	-100%	0.162
Ciprofloxacin 200 mg	126	9	2557.8	60.9	11.68 ± 61.79	0.85 ± 9.29	-92.86%	0.034*
Acyclovir 250 mg	1164	228	59,946	11,742	273.73 ± 990.26	54.87 ± 392.01	-80.41%	< 0.001*
Meropenem 1 gm	973	268	136,220	37,520	622.01 ± 1205.67	175.33 ± 578.62	-72.46%	< 0.001*
Linezolid 200 mg	150	55	3600	1320	16.44 ± 121.13	6.17 ± 90.23	-63.33%	0.062
Vancomycin 1 gm	810	347	51,030	21,861	233.01 ± 442.03	102.15 ± 298.61	-57.16%	< 0.001*
Ceftazidime 1000 mg	71	40	1008.2	568	4.60 ± 34.49	2.65 ± 28.24	-43.66%	0.430
Clindamycin 300 mg	74	57	1998	1539	9.12 ± 102.85	7.19 ± 56.29	-22.97%	0.247
Cefepime 1000 mg	50	40	1050	840	4.79 ± 48.08	3.93 ± 50.72	-20%	0.672
Ceftriaxone 1 gm	1130	1000	14181.5	12,550	64.76 ± 112.09	58.64 ± 92.37	-11.5%	0.910
Amikacin 500 mg	24	22	210	192.5	0.96 ± 7.19	0.90 ± 9.88	-8.33%	0.169
Colistin 100,000 IU	44	45	6160	6230	28.13 ± 218.43	29.11 ± 314.08	2.27%	0.502
Ampicillin Sulbactam 750 mg	152	158	1292	1343	5.90 ± 23.44	6.28 ± 33.09	3.95%	0.214
Cefoperazone Sulbactam1.5 gm	342	374	5985	6545	27.33 ± 96.89	30.58 ± 149.70	9.36%	0.097
Levofloxacin 500 mg	34	52	605.2	925.6	2.76 ± 15.94	4.33 ± 26.42	52.94%	0.261
Cefotaxime 1 gm	202	357	2464.4	4355.4	11.25 ± 46.81	20.35 ± 46.54	76.73%	< 0.001*
Total	5509	3033	313947.12	107592.4	1433.55 ± 2255.82	503.34 ± 862.22	-44.94%	< 0.001*

SD: Standard deviation U: Mann Whitney test p: p value for comparing between the studied phases

Sig.: Significance *: Statistically significant at *p* ≤ 0.05

**Fig. 4:**
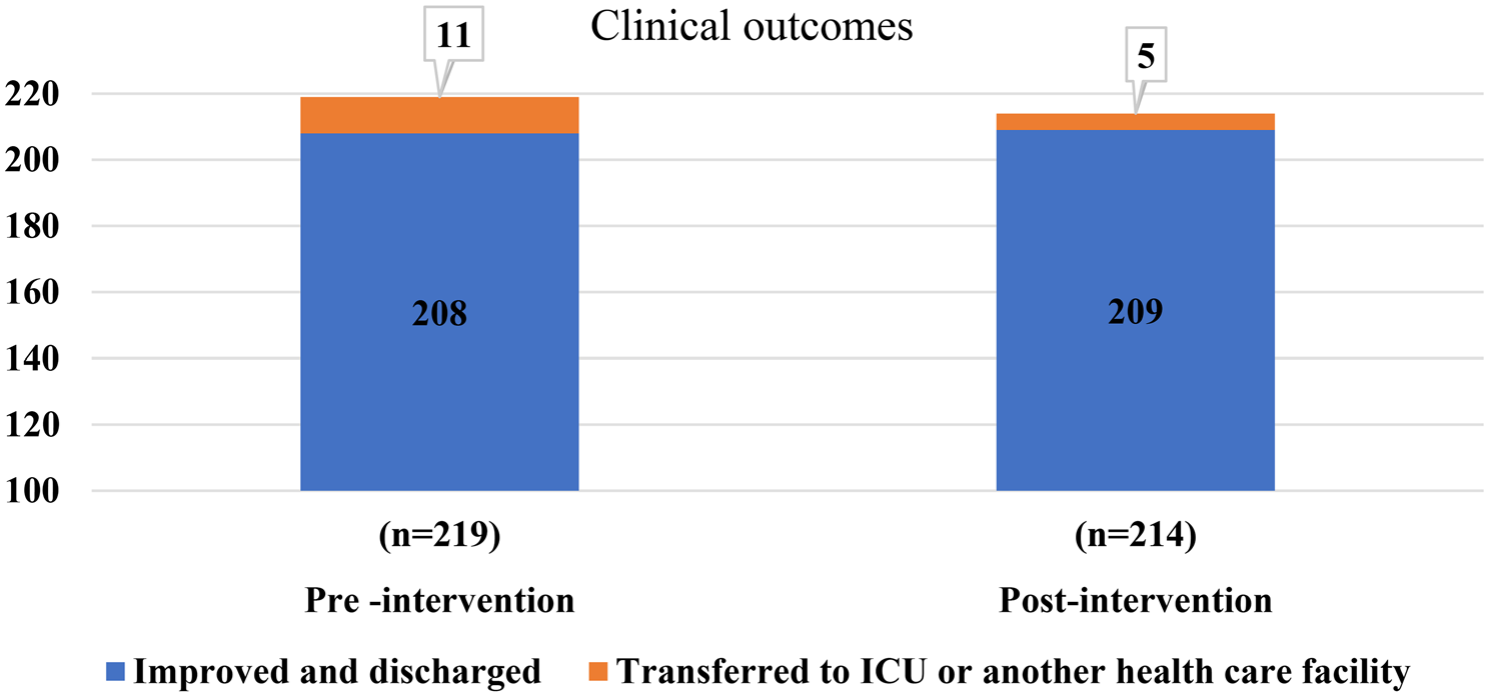
Clinical outcome of cases in the pre-and post-intervention phases

The outcome of the studied cases was almost identical: 98.6% (208/211) of patients demonstrated clinical improvement and were successfully discharged in the post-intervention phase, compared to 97.7% (209/214) in the pre-intervention phase. In the postintervention phase 2.3% (*n* = 5) of cases required transfer to the PICU (Fig. 4). None of studied children experienced deterioration of their medical conditions following de-escalation of antibiotic. These findings indicate that the ASP interventions significantly reduced costs and time without compromising patient safety or recovery.

## Discussion

Antimicrobials are the most common prescribed medications in Pediatrics; estimates show that between 37% and 61% of hospitalised children receive antibiotics during their hospital stay. Nevertheless between 20% and 50% of these prescriptions are deemed to be unnecessary or inappropriate [9]. It is well-known that the irrational use of antibiotics is the primary drive of antimicrobial resistance, creating a significant challenge for clinicians and call for the urgent implementation of Antimicrobial Stewardship Program (ASP) [10]. 

Implementing Antimicrobial Stewardship Program (ASP) in low- and middle-income countries is particularly challenging due to unique healthcare pressures. Because infections are a leading cause of mortality in children under five, clinicians often prescribe antibiotics at high rates. This issue is further complicated by a lack of diagnostic resources; without reliable microbiological testing or susceptibility data, healthcare providers frequently rely on broad-spectrum antibiotics to cover all possibilities, leading to significant overprescription [11]. Beyond the clinical and diagnostic barriers, several barriers exist. This includes shortage of trained pediatric infectious diseases specialist or antibiotic stewardship, a lack of governmental support, and the absence of standardized national protocols. Furthermore, a general lack of education regarding antibiotic use is exacerbated by a hierarchy where senior clinicians may not prioritize or recognize the necessity of stewardship efforts [12, 13]. This study represents the initial formal evaluation of an Antimicrobial Stewardship Program (ASP) at our facility. It specifically investigates the efficacy of a dual-strategy approach: the implementation of standardized clinical protocols paired with a prospective audit and feedback (PAF) model.

In the post-intervention phase, antibiotics prescribing demonstrated 70.6% adherence to the institutional guidelines. The high level of compliance aligns with findings by Bassiouny et al., who reported an 86% adherence rate in Cairo [14]. These results highlight the importance of establishing local guidelines especially in low and middle- income countries where there are limitations in microbiological diagnosis. In the current study, 29.4% (*n* = 63) of cases required a total of 70 interventions. The Antibiotic Time Out (ATO) was the most common intervention (41.4%), followed by addressing inappropriate prescriptions (28.6%) with a physician’s acceptance rate of 59%. The antibiotic time out was guided mainly by the availability of diagnostic information and culture results, and clinical response to therapy to assess continued need for antibiotics. Therefore, delay in culture results without symptom relief lowered the acceptance rate by physicians and led to unnecessary prolongation of the duration of empirical therapy. Similar findings were also detected by Klatte et al. [15] where 37.1% of the population studied required intervention with a higher acceptance rate (74.6%). They also reported that 45% of required intervention was antibiotics discontinuation, with a 64% acceptance rate. Furthermore, Li et al. [16] in China accessed the role of pharmacist in ASP and reported a higher acceptance rate (71.9%) with antibiotic discontinuation and de-escalation were the most common interventions. Similar results with high acceptance rate (79%) also detected in study in PICU in Germany with de-escalation and stop of antibiotic were the most common interventions [14]. 

Lower agreement (40.2%) was detected in Nigeria [18]. These findings emphasize the necessity of combining evidence-based guidelines with prospective audit-with-feedback in resource-limited countries. Improving clinician understanding of antibiotic spectrums may aid in increasing the acceptance rate. However, the relatively low compliance observed in this study could be attributed to senior consultants valuing their own professional judgment and fearing a compromise in clinical autonomy. Moreover, the clinicians’ agreement to the prospective audit and feedback was voluntary, which does not guarantee a high acceptance rate.

In the present study, the core achievement of the ASP was the facilitation of prompt antibiotic de-escalation once the microbiological data were available, regardless of the initial clinical indications. The specific types of infections may influence the initial selection of empiric therapy, but they do not sufficiently explain the significant 40.91% reduction in total Days of Therapy (DOT) observed in the post-intervention phase. Specifically, The Days of Therapy per 1000 (DOT/1000 PT) decreased significantly from 1545.52 to 1115.53 DOT/1000 PD, representing a 27.82% decrease in DOT/1000 PT. Similar findings were detected by Renk et al. [17] in the PICU, with an 18% decrease in DoT/1000 PD in the post-implementation period. Additionally, Zequinao et al. in Brazil [19] and Klatte et al. in Germany [15] reported declines in DOT/1000 PD (24.8% and 11.33%, respectively).

Differences in the reduction between different studies could be explained by differences in the consumption of antibiotics in pre and post implantation phase. In the present study there was a high consumption of antibiotics in both phases which could explain the small difference. Contrary; Kitano et al. [20] reported a higher percentage of reduction (76.2%) and this was explained by the assessment of antibiotics following culture results on holidays with early de-escalation of antibiotics, also a high baseline consumption of antibiotics in pre-intervention phase reduced following the implementation of ASP recommendations and prospective audit in the post intervention phase, and this was consistent with the present study.

In the present study there was significant reduction in tigecycline consumption from 14.17 to zero DOT/100PD (*P* = 0.001). Aiesh et al. detected a similar decline, but with a lower proportion of 62.08% [21]. Moreover, the DOT/1000 PT of meropenem decreased significantly (p = < 0.001). Aiesh et. A l [21] found similar results, with meropenem being the most frequently used antibiotic prior to ASP and decreased by 38% following ASP. Meropenem consumption fell in Germany (50%) [17], Tokyo [22] (59.3%), and Colorado (22.2%) [23]. 

The changes in antibiotic consumption in the present study could explained by first, meropenem was discontinued whenever there was no proof of extended spectrum beta lactams inhibitor (ESBL) or multidrug resistant organisms. Second, improvement of microbiological diagnosis by introducing molecular diagnoses using respiratory and cerebrospinal fluid multiplex PCR in the post intervention phase help in rapid and appropriate identification of causative pathogens in suspected CNS and respiratory infections which aids in early modification of empiric antibiotics with deescalation of antibiotics [6]. lastly, the use of the 3rd generation cephalosporin was significantly increased in postintervention phase which could reflect the adherence to the guidelines, as the common indications for antibiotics prescription in the post-interventions phase were lower respiratory tract infections and UTI, for which the 3rd generation cephalosporin is the empiric therapy.

In the present study a considerable reduction in vancomycin consumption [329.08 versus138.90 DOT/1000 patient’s days, *P* < 0.001] was observed, which was consistent with previous studies [17, 23]. This reduction in the vancomycin consumption, in addition to reflecting the benefit of ASP implementation, could also be explained by the high prevalence CNS infection (27.3%) in the pre-intervention phase versus 10.7% in the post-intervention phase. Despite the variations in the indications of antibiotic prescription, the primary success of ASP was in the timely de-escalation of therapy once the microbiological results released. Moreover, the variability in the indications could explain the initial drug choice, but it does not fully account for the 40.91% reduction in total Days of Therapy (DOT). The length of stay (LOS) is an indirect indicator of reduced health-care cost and infection risk. A systematic analysis conducted by Nathwani et al. [24] detected that 86% of the studies showed either a decrease or no change in LOS following the implementation of ASP, however there was a statistically significant difference and an average reduction in LOS of nearly 3.24 days was also detected.

Following ASP implementation, the average length of hospital stay reduced significantly (*P* < 0.001) from 12.8 to 10.8 days. Similarly, Bassiouny et al. [14] and Horikoshi et al. [22] observed a reduction in LOS [15.5 to 11.94 days, and 20.6 to 18.6 days; respectively]. In contrast Kreitmeyr et al. [25] observed no significant change, and Lee et al. [26] reported an increase in the LOS in the PICU and NICU (from 3.4 to 3.6 days and from 26.7 to 35 days respectively). Differences in the length of stay between different studies could be explained by other contributing factors such as presence of comorbid condition, and severity of the diseases.

Following ASP implementation in the current study, there was a significant reduction (*P* < 0.001) in the cost of antibiotics by 44.94%. More reduction in the cost of prescribed antibiotics was demonstrated by Zequinao et al. [19] (53.62%) and Kreitmeyr et al. [25] (50%), while Renk et al. [17], Wassef et al. [27] Ceradini et al. [28], and reported lower rates (40%, 2%, and 19.7% respectively) following ASP adoption. Reduction of antimicrobial costs suggests that ASP has a good impact on cost reduction. Studies that showed increased costs or low percent reduction may be attributed to lower adherence to ASP recommendations, increased use of expensive narrow spectrum antibiotics which led to an increase in the total cost. Also, the reduction in the use of some antibiotics that are not expensive might be a factor [26]. 

The current study’s findings demonstrated that educational initiatives, as well as prospective audits and feedback effectively antibiotic consumption and increased adherence to the institutional guidelines. This was in lines with earlier research [11, 18, 29, 30]. 

### Limitation of the study

The current study had some limitations. First, it was applied in a single pediatric ward. Second, the short duration of the study did not address the change in the antibiogram following implementation of ASP, as well as the long-term impact of seasonal variation on antimicrobial consumption. Third, the study is limited by its quasi-experimental, before-after design. Without a concurrent control group, we cannot completely rule out the influence of unmeasured external factors or secular trends on antibiotic utilization. However, the immediate and substantial reduction in broad-spectrum antibiotic use following the implementation of ASP protocols combined with stable clinical outcomes suggests that the stewardship program was the primary driver of these changes. Lastly, as the study was conducted during the COVID-19 outbreak, the difference in the causative agents of infections may have been influenced by a drop in certain bacterial illnesses and a shift to viral pathogens.

## Conclusion

Antimicrobial stewardship (ASP) remains a potential tool for optimizing antimicrobial prescriptions, with observed associations suggesting improvements in hospital expenses and length of stay. In this study, the implementation of prospective audit and feedback, as well as institutional guidelines correlated with improved antibiotic prescription. Annual updates to the institutional guidelines based on local antibiogram, is critical for guiding empiric antimicrobial therapy, especially in low- and middle income- countries with limited diagnostic capacity.

Further research over an extended period and on large scale is strongly recommended to assess the long-term impact of seasonal variation and change in the microorganism’s causing infection on the ASP.

## Supplementary Information

Below is the link to the electronic supplementary material.

Supplementary Material 1

## Data Availability

Most data analyzed during this study are included in this published article. The underlying datasets used and/or analysed during the current study are available from the corresponding author on reasonable request.

## Abbreviations

AMR: Antimicrobial resistant
ASP: Antimicrobial stewardship program
UTI: Urinary tract infection
DOT: Days of therapy
ATO: Antibiotic time out
PICU: Pediatric intensive care unit
