# Supplementary material for: Implementation of an antimicrobial stewardship program in Alexandria University Children’s Hospital: an interventional study
Source: Ital J Pediatr. 2026 Apr 10;52:56. doi: 10.1186/s13052-026-02236-3 (PMC13069748; doi:10.1186/s13052-026-02236-3)
Supplement: Supplementary file 1 — Supplementary Material 1 [file 13052_2026_2236_MOESM1_ESM.pdf]

Alexandria University Children's Hospital

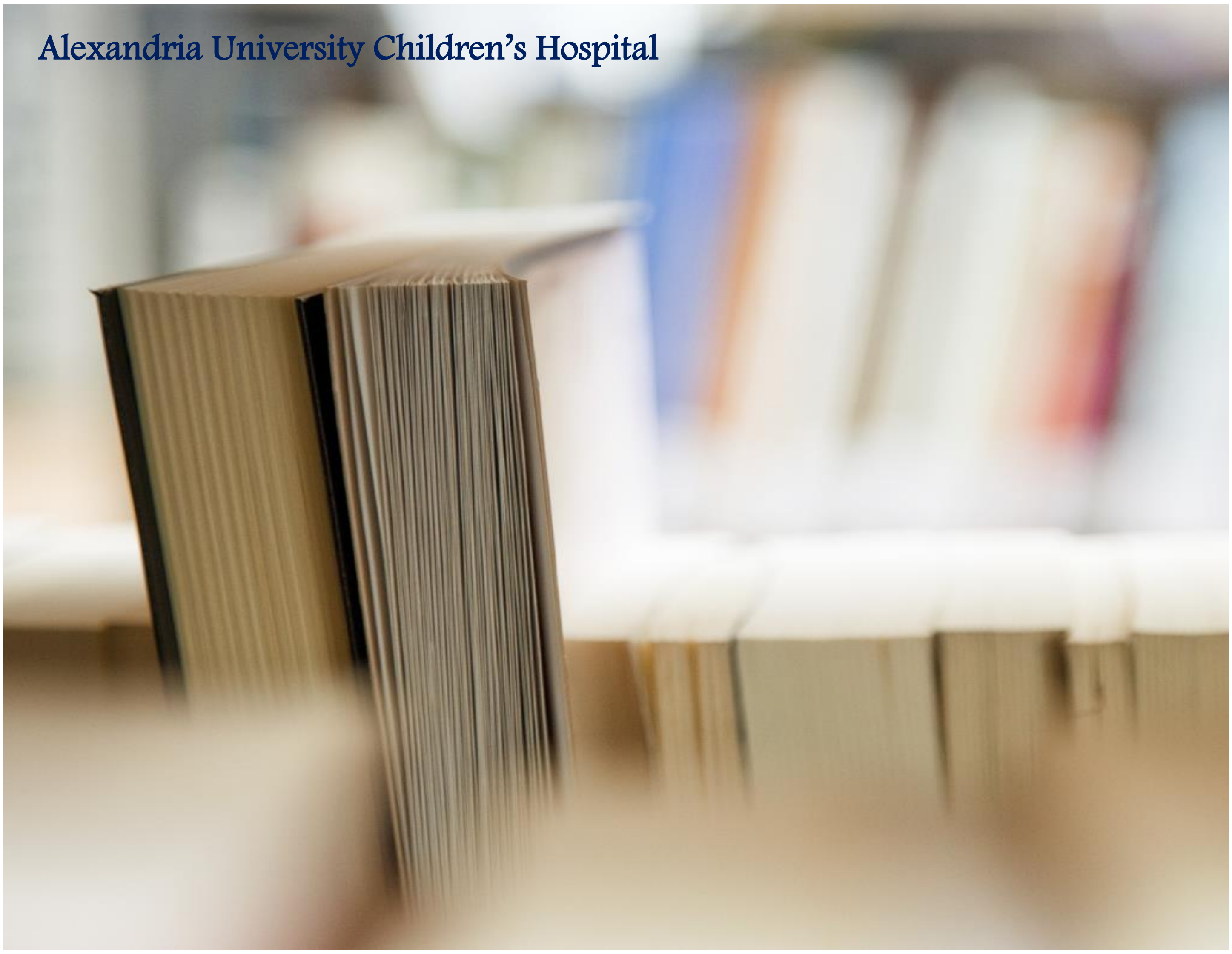

# EMPIRICAL ANTIMICROBIAL GUIDELINES

Based on local epidemiology and antimicrobial susceptibility patterns

# Acknowledgement

I would like to express my deep gratitude and appreciation towards all doctors and technicians working in the microbiology laboratory in Alexandria University Children hospital (AUCH) and Alexandria Main University hospital (AMUH) for their kind co-operation which helped me in completion of this guide.

They offered me all resources needed in running the program and integrating their laboratory results in data analysis for generating the hospital antibiogram, aiming at better interpretation and tailoring antimicrobial treatment.

I would like to thank them all for giving me such attention and time.

# This guide has been developed by:

**Marwa Ahmed Meheissen, MD**

**Assistant Professor of Microbiology and Immunology**

**Department of Microbiology and Immunology**

**Faculty of medicine**

**Eman Hamza Hassan, MD**

**Lecturer of Pediatrics**

**Pediatric Infectious diseases Unit**

**Faculty of medicine**

**Sarah El Sayed Saad, BCPS, Pharm D**

**Clinical pharmacist at AUCH**

**PhD candidate in microbiology at HIPH**

# Index

Preface ……………………………………………………………………………………4
List of Abbreviations……………………………………………………………………5
Community acquired pneumonia…………………………………………….…………6
Hospital acquired pneumonia…………………………………………………………...9
Bacterial meningitis……………………………………………………………………....12
Viral encephalitis…………………………………………………...……………………16
Infective endocarditis……………………………………………………………………19
Urinary tract infections…………………………………………………………………...22
Skin and soft tissue infections……………………………………………………………26
Well appearing febrile infants…...………………………………………………….……29
Severe sepsis and septic shock…………………………………………………………...30
Antibacterial drug dosages……………………………………………………………….32
Hospital antibiogram 2020/2021………………………………………………………....36

## Antibiotics Colour Guide

| | |
|---|---|
| Green | 1st line |
| Yellow | 2nd line /upgrade |
| Red | Add on |

# Preface

This guide is designed for use by medical professionals involved in curative care at the hospital.

We have tried to respond in the simplest and most practical way possible to the questions and problems faced by the medical staff, using the recommendations of reference organizations such as the World Health Organization (WHO) and American Academy of Paediatrics (AAP).

This edition touches on the curative aspects of the main infectious diseases encountered. The list is incomplete but covers the essential needs.

Our aim is to guide for the rational use of antimicrobials, decrease the pace to antimicrobial resistance and protect our scarce resources.

The guide is a collaborative effort of many disciplines; medical, microbiological and pharmaceutical field experiences.

The data concerning the hospital antibiogram is gathered from the microbiology laboratory through the year 2020 -2021.

Despite all efforts, it is possible that certain errors may have been overlooked in this guide. Please inform the authors of any errors detected.

# List of abbreviations

| | |
|---|---|
| **AAP** | American academy of pediatrics |
| **CAP** | Community acquired pneumonia |
| **CBC** | Complete blood count |
| **CFU** | Colony forming unit |
| **CNS** | Central nervous system |
| **CRP** | C- reactive protein |
| **CRE** | Carbapenem resistant Enterobacteriaceae |
| **CSF** | Cerebrospinal fluid |
| **CXR** | Chest X-ray |
| **DRESS** | Drug rash with eosinophilia and systemic symptoms |
| **EEG** | Electroencephalogram |
| **ESBL** | Extended spectrum beta-lactamase |
| **ESR** | Erythrocyte sedimentation rate |
| **FDA** | Food and Drug Administration |
| **GCS** | Glasgow Coma Scale |
| **GNR** | Gram negative rods |
| **HAP** | Hospital acquired pneumonia |
| **HSV** | Herpes simplex virus |
| **ICU** | Intensive care unit |
| **I&D** | Incision & drainage |
| **IE** | Infective endocarditis |
| **INR** | International normalized ratio |
| **IV** | Intravenous |
| **LP** | Lumbar puncture |
| **MDRO** | Multidrug resistant organism |
| **MRSA** | Methicillin-resistant Staphylococcus aureus |
| **PCR** | Polymerase chain reaction |
| **PO** | Per Os |
| **PT** | Prothrombin time |
| **PTT** | Partial thromboplastin time |
| **SIRS** | Systemic inflammatory response syndrome |
| **TEN** | Toxic epidermal necrolysis |
| **TMP/SMX** | Trimethoprim/ Sulfamethoxazole |
| **UTI** | Urinary tract infection |
| **VRE** | Vancomycin resistant enterococci |
| **VZV** | Varicella zoster virus |
| **WBC** | White blood count |

# Community acquired pneumonia (CAP)

It is defined as an acute pulmonary parenchymal infection in a patient who has not been hospitalized in the preceding 14 days .

## Severity of Pneumonia

| **Mild Pneumonia**<br>(Outpatient Treatment) | **Moderate Pneumonia**<br>(Inpatient Treatment) | **Severe Pneumonia**<br>(ICU Treatment) |
|---|---|---|
| • Age > 3 months<br>• **Absence of**:<br>o Retractions<br>o Grunting<br>o Nasal flaring<br>o Apnea<br>• Pulse oximetry > 92% in room air<br>• Non-toxic appearance<br>• Ability to tolerate oral medications and fluids<br>• Adequate observation/ follow-up care | • Age < 3 months<br>• Moderate-severe respiratory distress<br>o Retractions<br>o tachypnea<br>o Grunting<br>o Nasal flaring<br>o Apnea<br>• Pulse oximetry < 90% in room air<br>• Altered mental status<br>• Moderate to large parapneumonic effusion<br>• Concern for inadequate outpatient care/observation/follow-up<br>• Dehydration, vomiting, or inability to take oral medication<br>• Failure of initial outpatient treatment | • Signs of impending respiratory failure (lethargy, increasing work of breathing, and/or exhaustion with or without hypercarbia).<br>• Need for mechanical ventilator support.<br>• Signs of inadequate perfusion (change in mental status, hemodynamic instability).<br>• Recurrent apnea or slow irregular respirations.<br>• Parapneumonic effusion requiring emergent drainage. |

**Investigations needed in hospitalized children:**

- Chest X ray
- US chest in cases suspected to have effusion or empyema.
- CBC/diff, CRP
- Renal function tests for children receiving IV fluids.
- Microbiological diagnosis: sputum culture (or induced sputum), or nasopharyngeal aspirate (for viral infections) and blood culture (if effusion/empyema suspected).

**Respiratory rate normal ranges according to age**

| **Age** | **Respiratory rate** (breaths/min) |
|---|---|
| 0-2 months | 60 |
| 2-12 months | 50 |
| 1-5 years | 40 |
| > 5 years | 20 |

## Empirical antimicrobial guidelines

# Treatment of CAP according to suspected pathogens and severity

**Most common pathogens:**

Viruses (the majority), *Streptococcus pneumoniae Hemophilus influenzae.*
**Mycoplasma pneumoniae* & *Chlamydophilia pneumoniae* (in school age (older than 5 years)

**Moderate**

**Yes**

**Start Ampicillin/sulbactam or Ceftriaxone or Cefotaxime**

**+**

**Azithromycin/Clarithromycin**
(In children older than 5 years)

**No**

IF → **Severe**

**Refer to ICU guidelines**

### IF ASPIRATION

- **Start Ampicillin/sulbactam**
  **or**
- **Clindamycin**

- oral anaerobes are a possible pathogenic agent

### IF COMPLICATED PNEUMONIA

- **Start Ceftriaxone or Cefotaxime**
  **+**
  **Clindamycin**

- Effusion, empyema, necrotizing pneumonia , abscess

### IF INFLUENZA PNEUMONIA (see footnote p.7 )

- **Add Oseltamivir**

- It is recommended for children hospitalized with presumed influenza pneumonia or if the child is hospitalized during influenza season.
- Ideally started within 48 hours, after symptom onset

**IF PENICILLIN ALLERGY (see footnote p.7)**

**Substitute with Levofloxacin or Moxifloxacin**

**Risk factors for Pneumonia caused by MRSA (Add clindamycin)**

Known MRSA colonization, previous MRSA infection, recent hospitalization, antibiotic use ( receipt of IV antibiotics in the prior 3 months), recent influenza-like illness, necrotizing or cavitary pneumonia, empyema and immunosuppression

**Antibiotic time out**

The clinical condition of appropriately treated is expected to improve within 48 to 72 hrs. Consider **Treatment Failure. In the following :-** increasing respiratory distress, need for respiratory support or worsening fever curve after > 48 hours of first-line therapy at appropriate dosing.

## Footnotes:

### 1. Clinical suspicion of influenza pneumonia

During the **influenza season**, influenza virus infection should be considered (regardless of influenza immunization status or previous episode of influenza infection during the current season) in :

- ● Febrile child with acute onset of respiratory illness (even if these symptoms develop during hospitalization)
- ● Febrile child with exacerbation of underlying chronic pulmonary disease (eg, asthma, cystic fibrosis)
- ●Children with CAP.
- ●Child presents with fever (≥37.8°C) and cough, sore throat, or both in the absence of another known cause of illness when influenza virus is known to be circulating in the community.

Strongly consider testing all patients with CAP for viral causes of pneumonia during respiratory viral season.

### 2. Penicillin/cephalosporin allergy

Assess the nature of the penicillin allergy per the penicillin allergy pathway. Severe penicillin allergy includes any of the following: anaphylaxis, angioedema, cardiac arrest, respiratory distress, severe cutaneous reaction (for example, Stevens-Johnson syndrome, erythema multiforme, DRESS and TEN). Patients who experience hives or other mild allergic reaction with penicillin/amoxicillin can still receive cephalosporins.

**Clindamycin allergy**: In the rare event of a clindamycin allergy (for example, hives or anaphylaxis), vancomycin can replace clindamycin.

**Vancomycin allergy**: most vancomycin allergies are consistent with red man syndrome, which is a drug reaction that can be managed by **slowing** the vancomycin infusion time and considering **pre-medication** with diphenhydramine.

### 3. Atypical pneumonia

(Often characterized by non-lobar, patchy, or interstitial pattern on CXR; insidious onset; low-grade of fever, malaise, cough; and minimal auscultatory findings relative to CXR. It is often caused by respiratory viruses, and atypical bacterial pathogens including Mycoplasma pneumoniae and Chlamydia pneumoniae. Most atypical pneumonia is mild and self-limited; however, for disease requiring hospitalization, consider diagnostic testing and treatment as above.

# Hospital acquired pneumonia (HAP)

It is a type of pneumonia when symptoms develop ≥48 hours after hospital admission in a non-ventilated child.

**Most common pathogens include:**

**Early onset:** symptoms develop <5 days after admission and it is caused by susceptible organisms.
**Late onset:** symptoms develop ≥5 days after admission and it is caused by multi-drug-resistant organisms (MDRO) as ESBL Enterobacteriaceae, *Pseudomonas aeruginosa*, Acinetobacter, S. aureus and anaerobes.

**Treatment of HAP**

- Mild / Moderate → **Ceftriaxone or cefotaxime**
- Severe / higher risk of infection with MDRO and **low risk** of MRSA (**MSSA covered**) → **Cefepime or Piperacillin/Tazobactam or Meropenem****
- Severe / higher risk of infection with MDRO and **high risk** of MRSA → **Cefepime or Piperacillin/Tazobactam or Meropenem**** **Add vancomycin**

 **Piperacillin/tazobactam:** the combination with vancomycin **may** be associated with increased risk of acute kidney injury, **monitor** serum creatinine.

** **Meropenem:** should be used **only if** extended-spectrum gram-negative rods or anerobic coverage in aspiration pneumonia is a consideration**.**

**Risk factors for Pneumonia caused by MRSA (Add vancomycin)**

Prior IV antibiotic use within 90 days, high prevalence of MRSA in the unit, or who are at high risk for mortality (e.g., need for ventilatory support, septic shock, and immunosuppression)

## Step-Down Therapy with Clinical Improvement For CAP

**When to switch?**

**When the patient become afebrile with improvement of respiratory effort for 24- 48 hours and able to tolerate oral intake**

| If initial empirical antibiotic treatment was: | Transition patient to following oral antibiotics: |
|---|---|
| **Ceftriaxone** | Amoxicillin-clavulanate<br>*** OR ***<br>Levofloxacin |
| **Clindamycin + Ceftriaxone** | Clindamycin |
| **Vancomycin + Ceftriaxone** | Levofloxacin<br>***AND***<br>IF HIGH SUSPICION FOR MRSA<br>Clindamycin |

## Step-Down Therapy with Clinical Improvement For HAP

De-escalate the initial antibiotic therapy by changing an empiric broad-spectrum antibiotic regimen to a narrower antibiotic regimen,or changing from combination therapy to monotherapy.

**Total duration**

**Uncomplicated cases**

- Combined parenteral and oral therapy is 7 to 10 days
- or continue oral therapy at least one week beyond resolution of fever

**Complicated cases**

- The duration is determined by the clinical response but usually a total of four weeks
- Two weeks after the patient is afebrile and has improved clinically.

## References:

1. Harris M, Clark J, Coote N, et al. British Thoracic Society guidelines for the management of community acquired pneumonia in children: update 2011. Thorax 2011; 66:ii1.
2. Bradley JS, Byington CL, Shah SS, et al. The management of community-acquired pneumonia in infants and children older than 3 months of age: Clinical practice guidelines by the Pediatric Infectious Diseases Society and the Infectious Diseases Society of America. Clin Infect Dis 2011; 53:e25.
3. Uyeki TM, Bernstein HH, Bradley JS, et al. Clinical Practice Guidelines by the Infectious Diseases Society of America: 2018 Update on Diagnosis, Treatment, Chemoprophylaxis, and Institutional Outbreak Management of Seasonal Influenzaa. Clin Infect Dis 2019; 68:e1.
4. Silvennoinen H, Peltola V, Lehtinen P, et al. Clinical presentation of influenza in unselected children treated as outpatients. Pediatr Infect Dis J 2009; 28:372.
5. Downes KJ, Cowden C, Laskin BL, Huang YS, Gong W, Bryan M, Fisher BT, Goldstein SL, Zaoutis TE. Association of acute kidney injury with concomitant vancomycin and piperacillin/tazobactam treatment among hospitalized children. JAMA pediatrics. 2017 Dec 1;171(12):e173219
6. American Academy of Pediatrics. Tables of antibacterial drug dosages. In: Red Book: 2018 Report of the Committee on Infectious Diseases, 31st ed, Kimberlin DW, Brady MT, Jackson MA, Long SS, (Eds), American Academy of Pediatrics, Itasca, IL 2018. p.914.
7. Downes KJ, Cowden C, Laskin BL, et al. Association of acute kidney injury with concomitant vancomycin and piperacillin/tazobactam treatment among hospitalized children. JAMA Pediatr 2017; 171:e173219.

# Bacterial meningitis

**Clinical findings**

**Bacterial meningitis: -** commonly manifests with fever, headache, and a stiff neck; it may progress rapidly to shock and death. However, manifestations may be subtle.

**Common manifestations include:**

**Infants** : Fever, hypothermia, bulging fontanel, lethargy, irritability, seizures, respiratory distress, poor feeding, and vomiting.

**Older children** : Fever, headache, photophobia, meningismus, nausea/vomiting, confusion, lethargy, irritability ,and postive signs of meningeal irritation.

**Evaluation**

**Laboratory testing :**

**Initial laboratory testing should include:**

Blood culture,CBC with differential and platelet count.

Inflammatory markers (CRP).

Serum electrolytes, BUN, creatinine, glucose.

PT, INR, and PTT (especially in patients with petechiae or purpura)

**Lumbar puncture (LP):**

LP should be performed in all children with suspected meningitis, unless there is a specific contraindication to LP.

**Contraindications to LP include**:

Cardiopulmonary compromise, clinical signs of increased intracranial pressure (e.g. papilledema, focal neurologic signs), skin infection over the site for LPand significant coagulopathy.

**Empiric antibiotics administered as soon as possible.**

**CSF should be sent for the following :**

Cell count and differential, glucose and protein concentration, Gram stain, and culture.

**Neuroimaging (eg, head CT):**

**Indications for neuroimaging before LP include**

Severely depressed mental status (coma), papilledema, focal neurologic deficit, history of hydrocephalus and/or presence of a CSF shunt, recent history of CNS trauma or neurosurgery

LP should be performed as soon as possible after neuroimaging is completed, provided that the imaging has not revealed any contraindications.

## CSF interpretation

| Investigation | Normal | Bacterial meningitis | Viral encephalitis | TB meningitis | Fungal meningitis |
|---|---|---|---|---|---|
| **Opening pressure** | 10-20% | High | Normal /high | High | High /very high |
| **Colour** | Clear | Cloudy | Perfectly clear | Cloudy yellow | Clear/cloudy |
| **Cells** | < 5 | High<br>100-50000 | Slightly increased<br>5-1000 | Slightly increased<br><500 | Normal/high<br>0-1000 |
| **Differential** | Lymphocytes | Neutrophils | Lymphocytes | Lymphocytes | Lymphocytes |
| **CSF /plasma glucose** | 50-60% | <40% | Normal /Low | Low /very low<br>< 30% | Normal/low |
| **Protein (g/L)** | < 0.45 | > 1 | Normal/high<br>0.5-1 | High/ very high<br>1-5 | Normal/high<br>0.2-5 |

**Empiric antibiotic regimens for bacterial meningitis**

**Usual empiric regimen**

Vancomycin **PLUS** one of the following:

Ceftriaxone OR Cefotaxime

(Also for Basilar skull fracture/ CSF leak and Anatomic defects )

**If CSF Gram stain shows GNR**

Add an aminoglycoside to the regimen in **Box 1**:

Gentamicin OR Amikacin

**Neutropenic patients with malignancy , penetrating head trauma, recent neurosurgery and/or medical device in place.**

Vancomycin **PLUS** one of the following:

Cefepime OR Ceftazidime OR Meropenem

**PLUS** an aminoglycoside: Gentamicin OR Amikacin

(*S. aureus* and gram-negative organisms are potential pathogens).

Optimal management also includes removal/replacement of the CSF drain or shunt, if present

**Impaired cell-mediated immunity (eg, chemotherapy patients)**

Add high-dose ampicillin to the regimen in **Box 1**

(*Listeria* is a potential pathogen )

**NB. In case of β-lactam Allergy**

**1st Line recommendation:** Substitute ceftriaxone/any group alternative with **meropenem** (≥ 3 months).

**2nd Line recommendation**: Substitute ceftriaxone with **levofloxacin** (≥ 6 months and < 5 years).

## Duration of empiric therapy

**Continue until the culture results become available**

**Culture results**

1. **Postive** → Modify according to the culture results
2. **Negative with CSF pleocytosis** → Based on clinical situation
3. **Negative without CSF pleocytosis** → Discontinue antimicrobial therapy if cultures remain sterile after 48 to 72 hours

## Definitive Therapy Recommendations for Confirmed Bacterial Meningitis with Known Organisms

The duration of antimicrobial therapy depends upon the causative organism and clinical course.

**Intravenous antibiotics are recommended for the full course of treatment.**

| Organism | Treatment duration |
|---|---|
| *Neisseria meningitidis* | 5-7 days |
| *Hemophilus influenzae* | 7-10 days |
| *Streptococcus pneumoniae* | 10-14 days |
| *Streptococcus agalactiae* (Group B *Streptococcus*) | 14-21 days |
| *S. aureus* | At least 14 days |
| Gram-negative bacilli | 21 days or a minimum of 14 days beyond the first sterile CSF culture |

## References:


1. CDC A. Case definitions for infectious conditions under public health surveillance.
2. Tunkel AR, Hartman BJ, Kaplan SL, et al. Practice guidelines for the management of bacterial meningitis. Clin Infect Dis 2004; 39:1267.
3. Kim KS. Bacterial meningitis beyond the neonatal period. In: Feigin and Cherry's Textbook of Pediatric Infectious Diseases, 8th, Cherry JD, Harrison GJ, Kaplan SL, et al (Eds), Elsevier, Philadelphia 2019. p.309.
4. Sheldon L Kaplan, MD . Bacterial meningitis in children older than one month: Treatment and prognosis. Uptodate This topic last updated: Nov 24, 2020.
5. Sommers LM, Hawkins DS. Meningitis in pediatric cancer patients: a review of forty cases from a single institution. Pediatr Infect Dis J 1999; 18:902.
6. Molyneux E, Nizami SQ, Saha S, et al. 5 versus 10 days of treatment with ceftriaxone for bacterial meningitis in children: a double-blind randomised equivalence study. Lancet 2011; 377:1837.

# Viral encephalitis

## Case definition of encephalitis:

**Major criterion (required)**

- ✓ Altered mental status (defined as decreased or altered level of consciousness, lethargy, or personality change) lasting >24 hours with no alternative cause.

**Minor criteria**

(2 required for possible encephalitis; ≥3 required for probable or confirmed encephalitis)

- ✓ Documented fever ≥ 38°c within the 72 hours before or after presentation.
- ✓ Generalized or partial seizures not fully attributable to a pre-existing seizure disorder.
- ✓ New onset of focal neurologic findings.
- ✓ CSF WBC count ≥ 5/mm
- ✓ Abnormality (new) of brain parenchyma on neuroimaging suggestive of encephalitis.
- ✓ Abnormality on EEG that is consistent with encephalitis and not attributable to another cause

CSF interpretation …see p.13

# Viral encephalitis

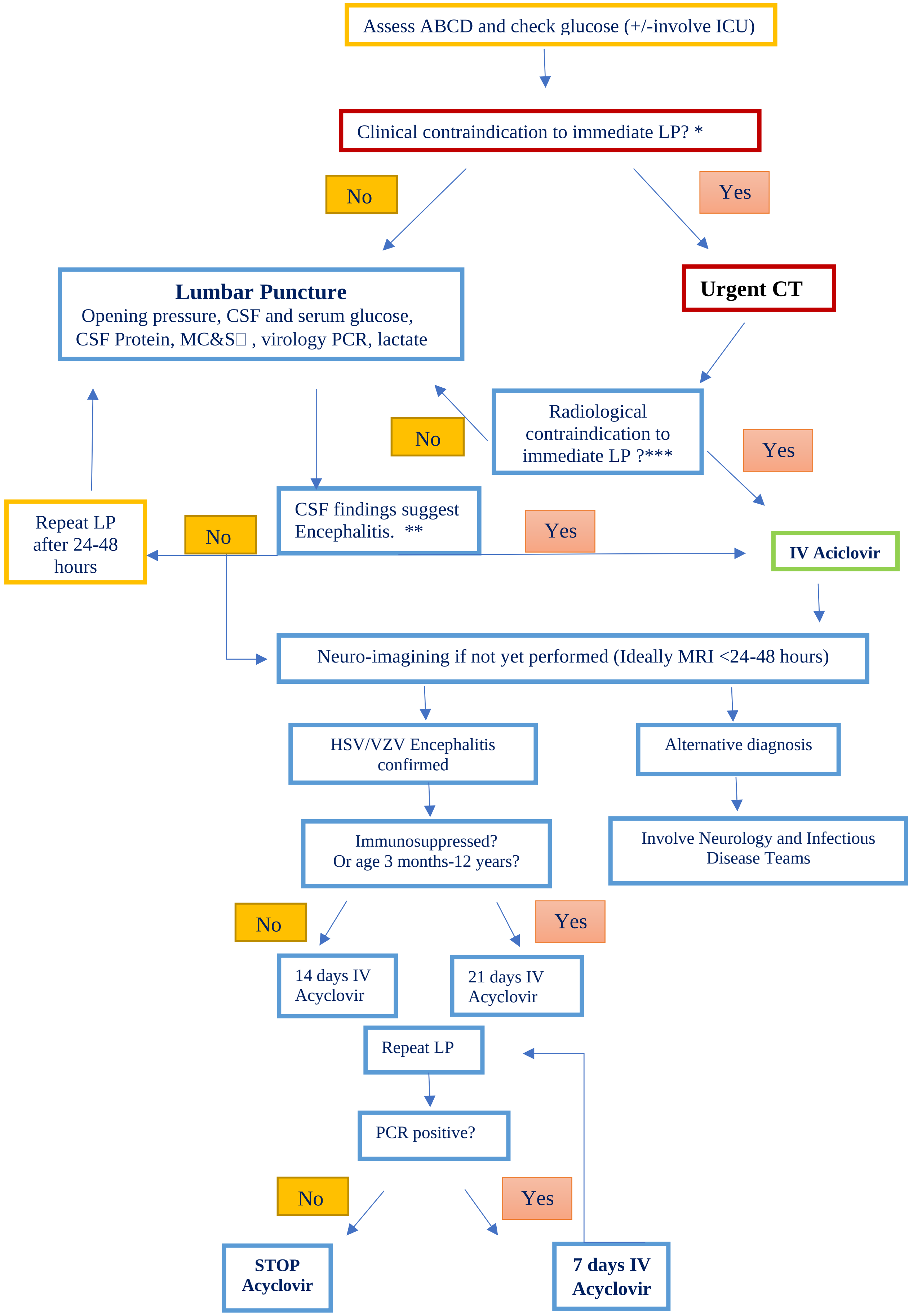

# Tables associated with the algorithm

*** Clinical contraindications to lumbar puncture without neuroimaging: -**

- Moderate to severe impairment of consciousness (GCS <13 or fall >2)
- Focal neurological signs (e.g., unequal, dilated or poorly responsive pupils)
- Abnormal posture or posturing
- Papilledema
- After seizures until stabilized
- Relative bradycardia with hypertension
- Abnormal 'doll's eye' movements Immunocompromise
- Systemic shock
- Coagulation abnormalities.
- Thrombocytopenia (Platelet count <100x10 /L)
- Local infection at lumbar puncture site
- Respiratory insufficiency

** **CSF interpretation** …see p.13

*** **Radiological Contraindications to LP**

Significant brain shift/swelling
Tight basal cisterns
Alternative diagnosis made

 **MC&S**: microscopy, culture and sensitivity

**References**:

1. Venkatesan A, Tunkel AR, Bloch KC, Lauring AS, Sejvar J, Bitnun A, Stahl JP, Mailles A, Drebot M, Rupprecht CE, Yoder J. Case definitions, diagnostic algorithms, and priorities in encephalitis: consensus statement of the international encephalitis consortium. Clinical Infectious Diseases. 2013 Oct 15;57(8):1114-28.
2. Solomon T, Michael BD (joint first), et al. On behalf of the National Encephalitis Guidelines Development Group. Management of suspected viral Encephalitis in adults: Association of British Neurologists and British Infection Association National Guideline. Journal of Infection 2012; 64(4):347-73.
3. Kneen R, Michael BD (joint first), et al. On behalf of the National Encephalitis Guidelines Development Group. Management of suspected viral Encephalitis in children: Association of British Neurologists and British Paediatric Allergy, Immunology and Infection Group National Guideline. Journal of Infection 2012; 64(5):449-77.

# Infective endocarditis

**IE**

**Definite IE**

**Pathologic criteria**

Pathologic lesions :Vegetation or intracardiac abscess demonstrating active endocarditis on histology, **OR**

Microorganism :Demonstrated by culture or histology of a vegetation or intracardiac abscess.

**Clinical criteria**

2 major clinical criteria, **OR** 1 major and 3 minor clinical criteria,**OR** 5 minor clinical criteria ...see table below.

**Possible IE**

Presence of 1 major and 1 minor clinical criteria **OR** presence of 3 minor clinical criteria

**Rejected IE**

A firm alternate diagnosis is made, **OR**

Resolution of clinical manifestations occurs after ≤4 days of antibiotic therapy, **OR**

No pathologic evidence of infective endocarditis is found at surgery or autopsy after antibiotic therapy for 4 days or less **OR**

Clinical criteria for possible or definite IE not met

## Clinical diagnosis of endocarditis

**Diagnostic score:**
2 major clinical criteria and 0 minor clinical criteria
1 major clinical criteria and 3 minor clinical criteria
0 major clinical criteria and 5 minor clinical criteria

| **Major clinical diagnostic criteria** | **Minor clinical diagnostic criteria** |
|---|---|
| 1. Positive blood cultures<br><br>For organisms that are typical* causes of IE: 2 positive cultures from samples drawn >12 hours apart; or<br>For organisms that are more commonly skin contaminants: 3 or a majority of ≥4 separate blood cultures (first and last sample drawn at least 1 hour apart).<br><br>2. Echocardiogram with (one or more of the following):<br><br>○ Vegetation (oscillating intracardiac mass on valve or supporting structures, in the path of regurgitant jets, or on implanted material in the absence of an alternative anatomic explanation)<br><br>○ Abscess<br><br>○ New partial dehiscence of prosthetic valve<br><br>○ New valvular regurgitation<br><br>3. Single positive blood culture for *Coxiella burnetii* or anti-phase 1 IgG antibody titer >1:800 | 1. Intravenous drug use or predisposing heart condition<br><br>Prosthetic heart valve or a valve lesion associated with significant regurgitation or turbulence of blood flow<br><br>2. Fever: Temperature >38.0°C (100.4°F)<br><br>3. Vascular phenomena (one or more of the following):<br><br>Major arterial emboli, Septic pulmonary infarcts, Mycotic aneurysm, Intracranial haemorrhage, Conjunctival haemorrhages, or Janeway lesions<br><br>4. Immunologic phenomena (one or more of the following):<br><br>Glomerulonephritis, Osler nodes, Roth spots, Rheumatoid factor<br><br>5. Microbiological evidence: Positive blood culture that does not meet major criteria (rare)** OR serological evidence of active infection with organism consistent with endocarditis |

*Typical microorganisms: Staphylococcus aureus, Viridans streptococci, Streptococcus gallolyticus (formerly S. bovis), coagulase negative staphylococci, HACEK group: Haemophilus, Aggregatibacter, Cardiobacterium, Eikenella, Kingella, enterococci.

**Rarer organisms: pneumococci, candida, g-ve bacilli, polymicrobial org.

In children who are **not acutely ill**, antibiotic therapy can be withheld for at least 48 hours while the blood cultures are collected.

**3 blood cultures** separate venipunctures → (Over 24 hrs) if no growth additional 2 blood cultures → (Over 24 hrs) result

**Laboratory tests supportive for the diagnosis of IE:**

- Low haemoglobin/haematocrit demonstrating anaemia (either haemolytic or anaemia of chronic disease).
- Elevated ESR and CRP.
- Urinalysis showing hematuria, proteinuria, and red cell casts is suggestive of glomerulonephritis, a minor diagnostic criterion.

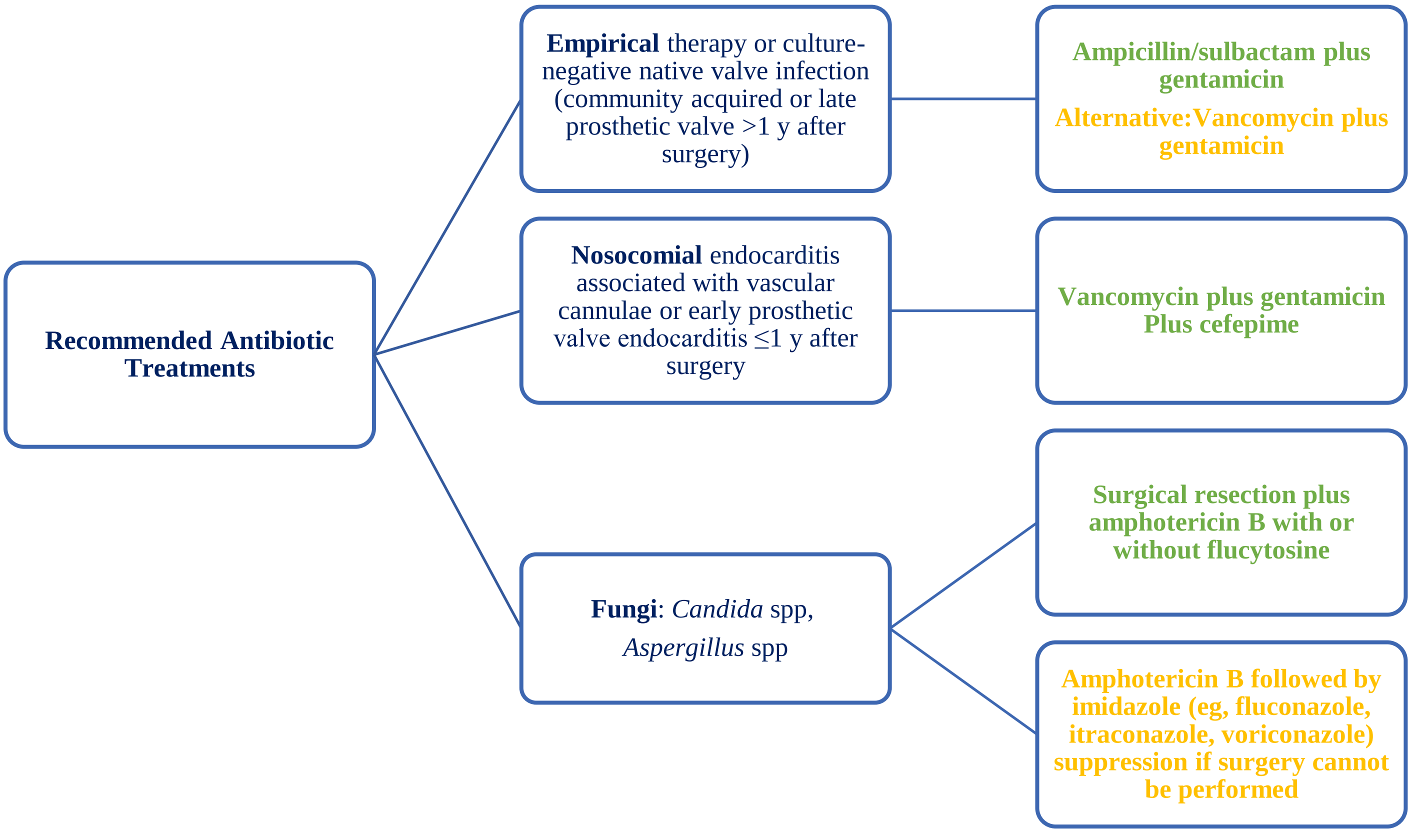


**N.B:**

- **For prosthetic valve endocarditis, add rifampin**
- **For definitive therapy: Antibiotic choice whether single or combination and duration of treatment is determined (according to your culture results and guidelines)**

## References


1. Baltimore RS, Gewitz M, Baddour LM, et al. Infective Endocarditis in Childhood: 2015 Update: A Scientific Statement From the American Heart Association. Circulation 2015; 132:1487.
2. Kavey RE, Frank DM, Byrum CJ, et al. Two-dimensional echocardiographic assessment of infective endocarditis in children. Am J Dis Child 1983; 137:851.
3. Penk JS, Webb CL, Shulman ST, Anderson EJ. Echocardiography in pediatric infective endocarditis. Pediatr Infect Dis J 2011; 30:1109.
4. Coward K, Tucker N, Darville T. Infective endocarditis in Arkansan children from 1990 through 2002. Pediatr Infect Dis J 2003; 22:1048.
5. Barbour SI, Louie EK, O'Keefe JP. Penetration of the atrialventricular septum by spread of infection from aortic valve endocarditis: early diagnosis by transesophageal echocardiography and implications for surgical management. Am Heart J 1996; 132:1287.
6. Karalis DG, Bansal RC, Hauck AJ, et al. Transesophageal echocardiographic recognition of subaortic complications in aortic valve endocarditis. Clinical and surgical implications. Circulation 1992; 86:353.
7. Amir G, Frenkel G, Rotstein A, et al. Urgent Surgical Treatment of Aortic Endocarditis in Infants and Children. Pediatr Cardiol 2019; 40:580.
8. Russell HM, Johnson SL, Wurlitzer KC, Backer CL. Outcomes of surgical therapy for infective endocarditis in a pediatric population: a 21-year review. Ann Thorac Surg 2013; 96:171.
9. Nishimura RA, Otto CM, Bonow RO, et al. 2014 AHA/ACC guideline for the management of patients with valvular heart disease: a report of the American College of Cardiology/American Heart Association Task Force on Practice Guidelines. J Am Coll Cardiol 2014; 63:e57.

# Urinary tract infections

## Assess symptoms and signs

**Infants and children presenting with unexplained fever ≥ 38°C should have a urine sample tested within 24 hours.**

| Age Group | Symptoms and Signs of UTI: Most Common to Least Common | | | |
|---|---|---|---|---|
| > 30 days – 18 years | Preverbal | Fever | Abdominal pain<br>Vomiting<br>Poor feeding<br>Flank pain / tenderness | Lethargy<br>Irritability<br>Hematuria<br>Malodorous urine<br>Failure to thrive |
| | Verbal | Frequency<br>Dysuria<br>Hesitancy<br>Urgency | Incontinence<br>Abdominal pain<br>Flank pain / tenderness | Fever<br>Malaise<br>Nausea<br>Vomiting<br>Hematuria<br>Malodorous urine<br>Cloudy urine |

- When infants and children with an alternative site of infection remain unwell, urine testing **should be considered after 24 hours**.

**Access risk factors and screening for UTI**

**Not toilet trained**

**Female Risk Factors**

T ≥ 39°C

Fever ≥ 2 days

No source of fever

< 12 months

**Number of Risk Factors Present**

Consider screening if ≥ 3

Recommend screening if ≥ 4

**Male Risk Factors**

Temperature ≥ 39°C

Fever ≥ 2 days

No source of fever

< 6 months

**Number of Risk Factors Present If Circumcised:**

Consider screening ≥ 3

Recommend screening ≥ 4

**IF uncircumcised:**

Consider screening ≥ 2

Recommend screening ≥ 3

**Toilet trained**

**All Patients**

Symptoms suggestive of to UTI

Prior history of UTI, fever ≥ 2 days

Prolonged fever (≥ 5 days)

Recommend screening **if any** of the above factors present

# Laboratory diagnosis of UTI

Positive leukocyte esterase or nitrite test
> 5 white blood cells per high-power field

Presence of bacteria on Gram stained urine

**Culture interpretation:**
**Clean catch sample:** $\geq 10^5$ (CFU)/mL
**Catheter sample:** $10^4$ -5 X $10^4$ CFU/mL
**Suprapubic sample:** $10^3$ CFU/mL

**Analysis if:**

Nitrite or leucocyte esterase positive: **LOW PROBABILITY UTI**

Nitrite and leucocyte esterase positive: **HIGH PROBABILITY UTI**

## Evaluation of the child with suspected urinary tract infection and initial urine with bacteriuria but no pyuria

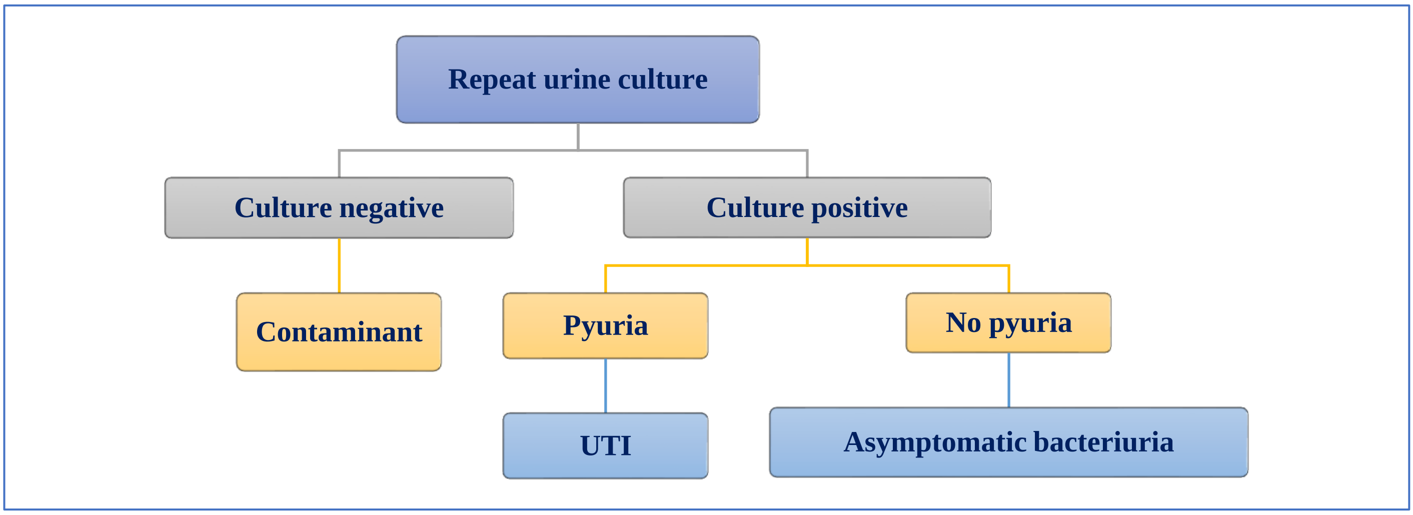

## Treatment

**Parentral therapy**

- Ceftriaxone
- Gentamicin /amikacin
- Cefoperazone

**Oral therapy**

- TMP/SMX
- Cefixime
- Cefdinir

➢ **Treatment duration:**

Parenteral route for 48 hours (for critically ill patients) and **switch to oral route** if clinical improvement occurs.

**Cystitis**:7–10 days as the total duration of therapy

**Pyelonephritis**: 7–14-day duration of therapy.

Follow-up routine urine culture: only after 48 hours if no clinical improvement, otherwise it is not recommended

Follow-up antibiotic prophylaxis: it is not recommended

## Fungal UTI

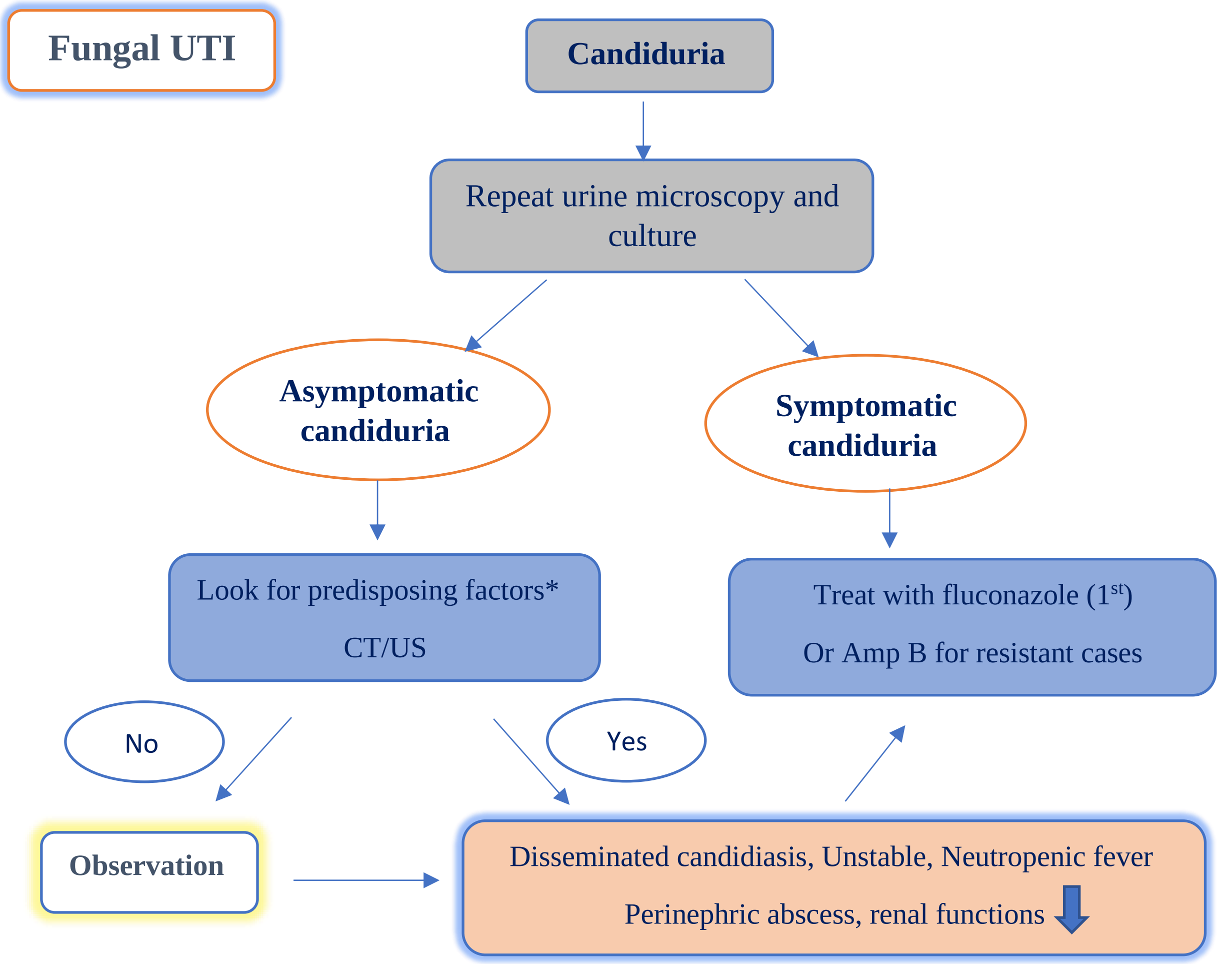


* Predisposing factors: urinary tract drainage devices, prior antibiotic therapy, diabetes, urinary tract pathology, and malignancy.

## References


1. SeattleHospital,Chang,P.Kaplan,R.,Ahn,J.,Brothers,A.,Fenstermacher,S.,McMahon,E.,Tang,E.,Tang,E., Migita,D.,2020 February.Urinary Tract InfectionPathway. Availablefromhttp://www.seattlechildren.org/pdf/UTI-pathway.pdf
2. Balighian, E. and Burke, M., 2018. Urinary tract infections in children.
3. Finnell SM, Carroll AE, Downs SM, the Subcommittee on Urinary Tract Infection. Technical Report--Diagnosis and Management of an Initial UTI in Febrile Infants and Young Children. Pediatrics 2011.
4. Fitzgerald A, Mori R, Lakhanpaul M. Interventions for covert bacteriuria in children. Cochrane Database Syst Rev 2012; 2:CD006943.
5. Pappas PG, Kauffman CA, Andes D, et al. Clinical practice guidelines for the management of candidiasis: 2009 update by the Infectious Diseases Society of America. Clin Infect Dis 2009; 48:503.
6. Thomas L, Tracy CR. Treatment of fungal urinary tract infection. Urologic Clinics. 2015 Nov 1;42(4):473-83.

# Skin and soft tissue infections

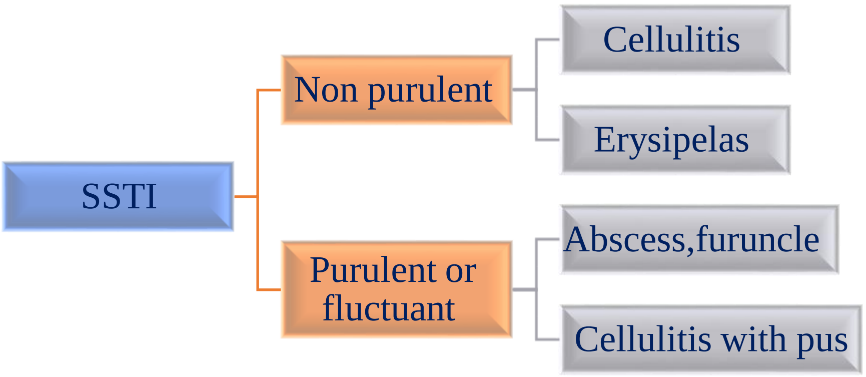


**SST infections are classified according to the severity into:**

| Type | Mild | Moderate | Severe |
|---|---|---|---|
| **Non purulent** | Afebrile | Febrile<br>No SIRS* see p.26 | Febrile with signs of SIRS*<br>Progression after 48 hours of oral therapy<br>Immunocompromised patients.<br>Infection near implanted devices<br>Clinical signs of deeper infection**<br>(e.g necrotizing fasciitis) |
| **Treatment** | **Oral**<br>Cephalexin<br>Clindamycin | Ampicillin/sulbactam<br>Ceftriaxone<br>Cefazolin<br>Clindamycin | Vancomycin<br>Plus<br>Piperacillin/Tazobactam |
| **Purulent** | Incision and drainage (I&D)<br>Mark the border of erythema<br>Follow up after 48 hours | I&D plus culture and sensitivity<br>Start IV antibiotic if:<br>• Extensive<br>• Poor compliance<br>• Follow up care is uncertain | I&D plus culture and sensitivity<br>Blood culture<br>Start empirical IV antibiotics against MRSA |
| **Treatment** | **Oral antibiotics**<br>Doxycycline¥<br>OR<br>TMP/SMX | **IV** Doxycycline ¥<br>OR<br>**IV** Clindamycin | **IV** Clindamycin<br>OR<br>**IV** Vancomycin<br>(If the child hemodynamically unstable). |
| **Duration** :5-7 days provided the patient improves clinically | | | |

**N.B.**

**Bullae, **or** skin sloughing **hypotension,** or organ dysfunction
¥ Tetracycline antibiotics may cause permanent tooth discoloration for children <8 years if used repeatedly. However, doxycycline binds less readily to calcium than other tetracycline antibiotics and may be used for ≤21 days in children of all ages (Red book 2018).

*Systemic inflammatory response syndrome (SIRS) defined by the presence of **two or more of the following criteria** (one of which must be abnormal temperature or leukocyte count):

| 1. **Core temperature** **>38.5°C or <36°C.** | 2. **Heart rate** **acc. to value by age** | | 3. **Respiratory rate** **acc. to value by age** | 4. **Leukocyte count** **acc. to value by age** |
|---|---|---|---|---|
| | (beats/minute) | | (breaths/minute) | ($x10^3/mm^3$) |
| **Age group** | **Tachycardia** | **Bradycardia** | | |
| **Infant (1 month to 1 year)** | >180 | < 90 | >34 | >17.5 or <5 |
| **Toddler and preschool (>1 to 5 years)** | >140 | NA | >22 | >15.5 or <6 |
| **School age (>5 to 12 years)** | >130 | NA | >18 | >13.5 or <4.5 |
| **Adolescent (>12 to <18 years)** | >110 | NA | >14 | >11 or <4.5 |

## Risk factors for community-associated and health care-associated methicillin-resistant *Staphylococcus aureus* infection in children and adolescents

### Community-associated MRSA infection

- Skin trauma (eg, "turf burns," lacerations, abrasions)
- Crowded living conditions
- Sharing potentially contaminated personal items (eg, towels)
- Challenges in maintaining personal cleanliness or hygiene
- Limited access to health care
- Frequent exposure to antimicrobial agents
- History of boils (furuncles), abscesses, or infection or colonization with MRSA in patient or close contact

### Health care-associated MRSA infection

- Invasive device
- History of health care-associated MRSA infection
- Surgery, hospitalization, dialysis, or residence in long-term care facility in previous 12 months
- Antibiotic use in the previous six months
- Tympanostomy tube for recurrent otitis media
- Age <2 years

## References:

1. Stevens DL, Bisno AL, Chambers HF, Dellinger EP, etal. Practice guidelines for the diagnosis and management of skin and soft tissue infections: 2014 update by the infectious disease's society of America. Clin Infect Dis. 2014;59(2):147.
2. Emr BM, Alcamo AM, Carcillo JA, Aneja RK, Mollen KP. Pediatric sepsis update: how are children different?. Surgical infections. 2018 Feb 1;19(2):176-83.
3. Goldstein B, Giroir B, Randolph A, International Consensus Conference on Pediatric Sepsis. International pediatric sepsis consensus conference: definitions for sepsis and organ dysfunction in pediatrics. Pediatr Crit Care Med. 2005;6(1):2.
4. American Academy of Pediatrics. Tetracyclines. In: Red Book: 2018 Report of the Committee on Infectious Diseases, 31st ed, Kimberlin DW, Brady MT, Jackson MA, Long SS (Eds), American Academy of Pediatrics, Itasca, IL 2018. p.905.
5. Sheldon L Kaplan, MD. Suspected Staphylococcus aureus and streptococcal skin and soft tissue infections in children >28 days: Evaluation and management. Jul 06, 2020. ed: UpToDate; 2020.

# Approach to well-appearing febrile infants 29 to 60 days of age

Fever more than 38°C with no apparent source of infection

Assess the risk for invasive bacterial infection *

-Complete sepsis evaluation**
-Hospitalization
-Start empiric antibiotics to cover E.coli and group B strept

**Ceftriaxone or Cefoperazone/sulbactam**

*** Risk factor for invasive bacterial infection:**

- ✓ Temp ≥ 38.6
- ✓ Incomplete immunization
- ✓ Prematurity
- ✓ Congenial or chromosomal defects
- ✓ Antibiotic therapy in the past 7 days
- ✓ Maternal factors:
  - Peripartum fever
  - Prolonged rupture of membranes
  - Positive vaginal culture for group B streptococcus

****Complete sepsis work-up:**

- CBC with differential
- CRP
- Blood culture
- Urine analysis and culture
- CSF analysis and bacterial culture
- CSF PCR for viral panel in case of CSF pleocytosis
- Chest X ray in presence of respiratory symptoms
- Stool culture in presence of diarrhea
- In case of high seasonal prevalence of influenza test for influenza

**Does Patient Meet Low Risk Criteria?**

- Non-toxic appearing
- Previously healthy term infant
- No focal bacterial infection apparent on exam
- WBC 5-15,000/mm3 and < 1500 bands/mm3
- Urinalysis: < 5 WBC/HPF and negative leukocyte esterase and nitrite
- Stool with negative blood
- Negative mucus: < 5 WBC/HPF stool (if done)
- CSF < 8 WBC/ul and negative Gram stain (if done).
- CXR negative (if done)

# Severe sepsis and septic shock

**RECOGNITION**

Fever or other evidence of infection (e.g.: petechial rash)

**And one or more** signs of impaired tissue perfusion:

Altered mental status, decreased urine output, tachycardia unrelated to fever, bradycardia, hypotension, cold shock, warm shock … **see box 1**

↓ **Vascular access**

CBC, peripheral blood culture, blood glucose, urea, electrolytes, creatinine, liver function tests, venous blood gas (to check lactate), coagulation profile.
**Correct hypoglycaemia** (if present) with 2ml/kg 10% glucose.

↓ **Within one hour**

**IV fluids**

20 ml/kg (60 ml/kg in 1st 30 mins)

**Antibiotics** …**see box 2**

**Fluid responsive**

**NO** → **Vasoactive drugs**

**YES** → **Admission for monitoring**

| Examination abnormalities | Cold shock | Warm shock |
|---|---|---|
| **Pulse** | Decreased | Bounding |
| **Capillary refill** | Delayed ≥3 sec | Flash <1 sec |
| **Skin** | Mottled, cool | Flushed, erythroderma |

**Box 1**

Community acquired sepsis…Ceftriaxone.

Intra-abdominal source… Ceftriaxone +Metronidazole or Piperacillin tazobactam or Carbapenem.

.

Immunocompromised or hospital acquired sepsis...Cefepime or Carbapenem or Piperacillin/tazobactam (Immunocompromised patients and preterm infants are also at risk of invasive fungal infections).

High prevalence of MRSA...Add Vancomycin.

Sepsis complicating an influenza-like illness...Add antiviral awaiting respiratory virus testing.

**Box 2**

**Empirical antimicrobial guidelines**

**References**:

1. Weiss SL, Peters MJ, Alhazzani W, Agus MS, Flori HR, Inwald DP, Nadel S, Schlapbach LJ, Tasker RC, Argent AC, Brierley J. Surviving sepsis campaign international guidelines for the management of septic shock and sepsis-associated organ dysfunction in children. Intensive care medicine. 2020 Feb;46(1):10-67.

2. Paul R. Recognition, diagnostics, and management of pediatric severe sepsis and septic shock in the emergency department. Pediatric Clinics. 2018 Dec 1;65(6):1107-18.

3. Goldstein B. International Consensus Conference on Pediatric Sepsis. International pediatric sepsis consensus conference: definitions for sepsis and organ dysfunction in pediatrics. Pediatric. Crit. Care Med. 2005; 6:2-8.

## Antibacterial Drugs for Paediatric Patients Beyond the Newborn Period

| Drug | Dose | Warnings & precautions<br>Adverse effects |
|---|---|---|
| **Amikacin** | 15–22.5 mg divided in 2–3 doses or in 1 dose | Higher doses than those given are appropriate for cystic fibrosis. |
| **Ampicillin/sulbactam** | 100–200 mg divided in 4 doses.<br>200–400 mg divided in 4 doses for meningitis | Max 8 g per day<br>Dosed on ampicillin component. |
| **Amoxicillin/clavulanic** | 4:1 Formulation:<br>20–40 mg divided in 3 doses, | Max 1500 mg per day.<br>Dosed on amoxicillin component. |
| **Azithromycin** | P.O: 5–10 mg once daily for the immediate release products.<br><br>I.V: 10 mg once daily | Per dose max: 250 mg for 6 mg/kg, 500 mg for 10–12 mg/kg,<br>1.5 g for 30 mg/kg.<br><br>Max 500 mg per day. |
| **Cefazolin** | 25–75 mg divided in 3 doses<br><br>Up to 150 mg divided in 3–4 doses for bone/joint infections | Max 12 g per day |
| **Cefotaxime** | 150–180 mg divided in 3 doses | Max 8 g per day |
| **Cefoxitin** | 80–160 mg divided in 3–4 doses. | Max 12 g per day<br><br>Active against anaerobes. |
| **Ceftriaxone** | 50–75 mg once daily.<br><br>100 mg divided in 1 or 2 doses. | Max 4 g per day.<br><br>Larger dosage appropriate for meningitis |
| **Ceftazidime** | 90–150 mg divided in 3 doses.<br><br>200–300 mg divided in 3 doses for serious *Pseudomonas* infections | Max 6 g per day. |
| **Cefoperazone** | 2:1 Formulations<br><br>60-80 mg/ kg /day divided in 2-4 divided doses | Dosed on cefoperazone component<br><br>Max dose of **sulbactam** in paediatrics **should not exceed** 80 mg /kg/day. |
| **Cefepime** | 100 mg divided in 2 doses<br>150 mg divided in 3 doses for *Pseudomonas* infections or febrile neutropenia | Max 4 g per day<br><br>Extended infusion may be needed for susceptible dose-dependent infections |

| | | |
|---|---|---|
| **Cefdinir** | 14 mg divided in 1 or 2 doses. | Max 600 mg/day |
| **Cefixime** | 8 mg divided in 1 or 2 doses | Max 400 mg per day |
| **Clarithromycin** | 10 mg once daily<br><br>PO 15 mg divided in 2 doses | Max 500 mg per day (IV)<br><br>Max 1 g per day (Oral) |
| **Clindamycin** | P.O: 10–25 mg divided in 3 doses<br>Severe: 30–40 mg divided in 3–4 doses<br><br>I.V: 20–40 mg divided in 3–4 doses | Max 1.8 g per day (Oral)<br><br>Max 2.7 g per day (IV) |
| **Ciprofloxacin** | P.O: 20–40 mg divided in 2 doses<br><br>I.V: 20–30 mg divided in 2 or 3 doses | Max 750 mg per dose (Oral)<br><br>Max 400 mg per dose (IV)<br><br>FDA recommends restricting use for certain uncomplicated infections because of serious adverse reactions. |
| **Colistimethate** | 2.5–5 mg base divided in 2–4 doses OR in terms of IU<br><br>Children ≤ 40kg<br>75,000-150,000 IU/kg/day divided in 3 doses. | Up to 7 mg base/kg/day may be required.<br><br>1 mg base = 2.7 mg colistimethate. |
| **Doxycycline** | 2.2–4.4 mg divided in 2 doses | Max 200 mg per day<br><br>The AAP stated that doxycycline can be administered for short durations (ie, 21 days or less) **without regard to the patient's age.**<br>Avoid excess sun exposure to avoid photosensitivity. |
| **Erythromycin** | 20 mg divided in 4 doses<br><br>Po: 40–50 mg divided in 3–4 doses | Max 4 g per day (IV and Oral).<br><br>Administer over at least 60 minutes to potentially prevent cardiac arrhythmias. |
| **Ertapenem** | 30 mg divided in 2 doses<br><br>≥13 y and adults, 1 g once daily | Max 1 g per day |
| **Gentamicin** | 6–7.5 mg divided in 3 doses, or<br>4.5–7.5 mg /dose every 24 hrs | Once daily dosing in patients with normal renal function |

| | | |
|---|---|---|
| **Imipenem/cilastatin** | 60–100 mg divided in 4 doses. | Max 4 g per day<br><br>Caution in use for treatment of CNS infections because of increased risk of seizures. |
| **Meropenem** | 60 mg divided in 3 doses<br><br>120 mg divided in 3 doses for meningitis. | Max 6 g per day<br><br>Extended infusion may be needed for susceptible dose-dependent infections. |
| **Levofloxacin** | ≥ 6 mo and < 5 yrs: 16-20 mg divided in 2 doses.<br><br>>5yrs: 10 mg/kg /day, max. 500 mg once daily dose | Max 500 mg per day<br><br>FDA recommends restricting use for certain uncomplicated infections because of serious adverse reactions |
| **Linezolid** | ≤11 y of age: 30 mg divided in 3 doses<br>>11 y of age: 1200 mg (not per kg) divided in 2 doses. | Myelosuppression increases with duration of therapy over 10 days. |
| **Metronidazole** | 22.5–40 mg divided in 3 or 4 doses<br><br>po:15–50 mg divided in 3 doses<br><br>30 mg divided in 4 doses for *C difficile* infection. | Max 4 g per day (IV)<br><br>Max 2.25 g per day (oral) |
| **Nitrofurantoin** | 5–7 mg divided in 4 doses<br><br>UTI prophylaxis: 1–2 mg once daily | Max 400 mg per day<br><br>For treatment of cystitis; not appropriate for pyelonephritis. |
| **Piperacillin/tazobactam** | 240–300 mg divided in 3–4 doses | Max 16 g per day<br><br>Dosed on piperacillin component. Extended infusion may be needed for susceptible-dose dependent infections. |
| **Rifampin** | 15–20 mg divided in 1–2 doses. | Should not be used routinely as monotherapy because of rapid emergence of resistance.<br>Max. 600 mg per day |
| **TMP/SMX** | 6-12 mg divided in 2 doses<br><br>2 mg once daily for UTI prophylaxis | Dosed on TMP component.<br><br>Max 160 mg per dose |

| | | |
|---|---|---|
| | 15–20 mg divided in 3–4 doses for *Pneumocystis jirovecii* treatment<br><br>5-10 mg divided in 2 doses, 3 times/wk for prophylaxis | |
| **Vancomycin** | 45–60 mg divided in 3–4 doses | Monitor serum concentration<br>Maximum 2000 mg/ day |

**References**

1. American Academy of Pediatrics. Tables of antibacterial drug dosages. In: Red Book: 2018 Report of the Committee on Infectious Diseases, 31st ed, Kimberlin DW, Brady MT, Jackson MA, Long SS, (Eds), American Academy of Pediatrics, Itasca, IL 2018. p.91
2. The Harriet Lane Handbook: A Manual for Pediatric House Officers, 22nd Edition 2020.
3. Wolters Kluwer | Lexicomp.

**Alexandria University Children Hospital**

# Hospital antibiogram 2020-2021

| Organism | Penicillin %S | Amoxicillin/clavulanate %S | Ampicillin %S | Amp/sulbactam %S | Cefuroxime %S | Ceftriaxone %S | Cefotaxime %S | Cefoxitin %S | Cefoperazone /sulbactam %S | Ceftazidime %S | Cefepime %S | Levofloxacin %S | Ciprofloxacin %S | Doxycycline %S | Fosfomycin %S | Amikacin %S | Gentamicin %S | Imipenem/cilastatin %S | Meropenem %S | Ertapenem %S | Piperacillin/tazobactam %S | Azithromycin %S | Erythromycin %S | Clindamycin %S | TMP/SXT %S | Rifampicin %S | Vancomycin %S | Teicoplanin %S | Linezolid %S | Colistin %S | Tigecycline %S | Nitrofurantoin %S |
|---|---|---|---|---|---|---|---|---|---|---|---|---|---|---|---|---|---|---|---|---|---|---|---|---|---|---|---|---|---|---|---|---|
| aba | 0 | 50 | 0 | 100 | 0 | 66.7 | 60 | 66.7 | 66.7 | 61.5 | 54.5 | 77 | 78 | 91.7 | 0 | 71.4 | 60 | 61.5 | 72.7 | 100 | 69.2 | 0 | 0 | 0 | 100 | 0 | 0 | 0 | 0 | 71.4 | 100 | 0 |
| pr | 0 | 0 | 50 | 50 | 50 | 100 | 0 | 0 | 0 | 50 | 50 | 0 | 50 | 100 | 0 | 100 | 100 | 100 | 100 | 100 | 100 | 0 | 0 | 0 | 0 | 0 | 0 | 0 | 0 | 100 | 0 | 0 |
| pae | 0 | 0 | 0 | 0 | 0 | 0 | 50 | 0 | 78.8 | 65 | 70 | 80 | 85 | 0 | 0 | 87 | 97 | 79 | 83.8 | 100 | 92.9 | 0 | 0 | 0 | 0 | 0 | 0 | 0 | 0 | 76 | 0 | 0 |
| cfr | 0 | 66.7 | 0 | 50 | 40 | 44.4 | 25 | 50 | 83.3 | 76.8 | 50 | 65 | 44.4 | 75 | 100 | 100 | 75 | 66.7 | 83.3 | 100 | 62.5 | 0 | 0 | 0 | 33.3 | 0 | 0 | 0 | 0 | 85.7 | 100 | 80 |
| ecl | 0 | 50.3 | 0 | 33.3 | 25 | 33.3 | 33.3 | 20 | 20 | 37.6 | 40 | 33.3 | 73 | 80 | 0 | 92 | 100 | 33.3 | 38 | 100 | 36 | 0 | 0 | 0 | 25 | 0 | 0 | 0 | 0 | 75 | 100 | 100 |
| eco | 0 | 67.6 | 16 | 69.6 | 26 | 29 | 29.4 | 78.3 | 67.7 | 76.8 | 50.3 | 61.6 | 60 | 73 | 91.7 | 100 | 73 | 82.3 | 80 | 100 | 69.7 | 0 | 0 | 0 | 41 | 0 | 0 | 0 | 0 | 89.7. | 90 | 95 |
| kpn | 0 | 50 | 0 | 50 | 16.7 | 26.9 | 23.1 | 33.3 | 100 | 27.6 | 37.6 | 75 | 64 | 87 | 0 | 77.8 | 60 | 60 | 50 | 50 | 42.9 | 0 | 0 | 0 | 61.5 | 0 | 0 | 0 | 0 | 95.7 | 100 | 50 |
| sau | 15 | 0 | 0 | 0 | 0 | 0 | 0 | 44.9 | 0 | 0 | 0 | 85.2 | 75.1 | 97.5 | 0 | 97.3 | 70.5 | 0 | 0 | 0 | 0 | 30.6 | 34 | 74.6 | 64.5 | 100 | 100 | 100 | 100 | 0 | 0 | 83 |
| scn | 33.3 | 0 | 0 | 0 | 0 | 0 | 0 | 60 | 0 | 0 | 0 | 100 | 100 | 83.3 | 0 | 100 | 100 | 0 | 0 | 0 | 0 | 50 | 60 | 0 | 100 | 100 | 100 | 100 | 100 | 0 | 0 | 0 |
| bsa | 100 | 0 | 100 | 0 | 0 | 100 | 100 | 0 | 0 | 0 | 100 | 0 | 0 | 0 | 0 | 0 | 0 | 0 | 0 | 0 | 0 | 0 | 100 | 0 | 0 | 0 | 100 | 100 | 100 | 0 | 0 | 0 |
| spn | 0 | 0 | 100 | 0 | 0 | 100 | 100 | 100 | 0 | 0 | 100 | 83.3 | 0 | 80 | 0 | 100 | 100 | 0 | 0 | 0 | 0 | 0 | 81.8 | 90 | 10 | 85.7 | 100 | 100 | 100 | 0 | 0 | 0 |
| svi | 83.3 | 0 | 100 | 0 | 0 | 66.7 | 57.1 | 0 | 0 | 0 | 85.7 | 100 | 0 | 100 | 0 | 0 | 0 | 0 | 0 | 0 | 0 | 0 | 80 | 83.3 | 0 | 100 | 87.5 | 85.7 | 100 | 0 | 0 | 0 |
| efa | 27.8 | 0 | 53 | 0 | 0 | 0 | 0 | 0 | 0 | 0 | 0 | 50 | 36.4 | 61.9 | 100 | 0 | 0 | 0 | 0 | 0 | 0 | 0 | 13 | 0 | 0 | 44 | 60.9 | 75 | 95.7 | 0 | 0 | 92 |

| Organism | Fluconazole %S | Amphotericin B %S | Voriconazole %S |
|---|---|---|---|
| Cal | 83.3 | 92 | 94 |

## Empirical antimicrobial guidelines

## ➢ Abbreviations

| Org | Organism |
|---|---|
| aba | Acinetobacter baumannii |
| Pr | Proteus sp. |
| pae | Pseudomonas aeruginosa |
| cfr | Citrobacter freundii |
| ecl | Enterobacter sp. |
| eco | Escherichia coli |
| kpn | Klebsiella pneumoniae |
| sau | Staphylococcus aureus |
| scn | Staphylococcus, coagulase negative |
| bsa | Streptococcus, beta-haem. Group A |
| spn | Streptococcus pneumoniae |
| svi | Streptococcus viridans, alpha-hem. |
| efa | Enterococcus sp. |
| cal | Candida sp. |

## ➢ Overall % susceptibility of antibiotics to be used for empiric treatment

| Amox/clav% | Amp/sulb% | Penicillin V % | Pip/tazobactam % | Azithromycin % | Cefepime % | Cefotaxime% | Ceftazidime % | Ceftriaxone % | Cefoperazone/sulb% | Erythromycin % | Fosfomycin % | Gentamicin % | Amikacin % | Levofloxacin % |
|---|---|---|---|---|---|---|---|---|---|---|---|---|---|---|
| 59.8 | 63.5 | 20.9 | 69 | 31 | 56.4 | 66.7 + | 97+ | 75 + | 97.7+ | 34.9 | 94.7 | 72.9 | 93.9 | 86.6 + |
| | | | | | | 31 - | 63.2- | 39.8 - | 65.2- | | | | | 77.5 - |

| Ciprofloxacin % | Clindamycin % | Vancomycin % | Teicoplanin % | Linezolid % | Nitrofurantoin % | Rifampin % | Tigecycline % | TMP/SXT % | Doxycycline% | Colistin % | Ertapenem % | Imp/cilastatin% | Meropenem % |
|---|---|---|---|---|---|---|---|---|---|---|---|---|---|
| 69.3+ | 75.6+ | 93.6 | 100 | 99.3 | 84.1 | 76.1 | 100 | 59.8 | 93.2+ | 87.7 | 90.9 | 72.9 | 71.4 |
| 74 - | | | | | | | | | 86.6- | | | | |

**N.B** **(+)** is % S for gram +ve **(-)** ...% S for gram -ve

**Commentary notes…**

- Susceptibility data are retrieved from WHONET software, developed and supported by the WHO Collaborating Centre for Surveillance of Antimicrobial Resistance, and used for the analysis of microbiology laboratory data with a focus on antimicrobial resistance surveillance.
- % Susceptible is calculated based on **non-resistant antimicrobials** (includes both the % intermediate and the % Susceptible).
- %MRSA is exceeding 20% in blood cultures, so anti MRSA is to be added for empiric treatment of blood stream infections and deescalated if MRSA risk is excluded to avoid resistance development.
- **% of high priority pathogens** according to WHO global priority pathogens list are:

**MRSA** 55% **VRE** 39% **ESBL** 71% **CRE** 44 %

# Main references

UpToDate. Evidence-based clinical decision support resource

Centres for Disease Control and Prevention

World Health Organization
